# The effects of ultrasound exposure on P-glycoprotein-mediated multidrug resistance in vitro and in vivo

**DOI:** 10.1186/s13046-018-0900-6

**Published:** 2018-09-19

**Authors:** Chixiong Huang, Senlin Huang, Hairui Li, Xinzhong Li, Bing Li, Lintao Zhong, Junfeng Wang, Meishen Zou, Xiang He, Hao Zheng, Xiaoyun Si, Wangjun Liao, Yulin Liao, Li Yang, Jianping Bin

**Affiliations:** 1 State Key Laboratory of Organ Failure Research, Department of Cardiology, Nanfang Hospital, Southern Medical University, 1838 Guangzhou Avenue North, Guangzhou, 510515 China; 2 Department of Gastroenterology, Guangdong Provincial Key Laboratory of Gastroenterology, Nanfang Hospital, Southern Medical University, Guangzhou, 510515 China; 3 Department of Oncology, Nanfang Hospital, Southern Medical University, Guangzhou, 510515 China; 4 Department of Pharmacy, Nanfang Hospital, Southern Medical University, Guangzhou, People’s Republic of China

**Keywords:** Ultrasound, Multidrug resistance, P-glycoprotein, Reactive oxygen species

## Abstract

**Background:**

Multidrug resistance (MDR) is often responsible for the failure of chemotherapy treatment, and current strategies for cancer MDR are not adequately satisfying as to their efficacy and safety. In this study, we sought to determine the anti-MDR effects of ultrasound (US) irradiation and its underlying mechanisms against drug-resistance.

**Methods:**

MDR variant MCF-7/ADR cell lines and endothelial cell lines were used to determine the appropriate ultrasound intensity for in vitro experiments. MCF-7/ADR cell and HEPG2/ADM cells were used to assess the anti-MDR effect of US irradiation. Intracellular adriamycin (ADM) accumulation, Cell viability, cell proliferation and cell apoptosis were evaluated after ADM + US treatment or ADM treatment alone. MCF-7/ADR xenograft mice were used to investigate the appropriate ultrasound intensity for in vivo experiments and its effect on the long-term prognosis. Underlining mechanisms by which ultrasound exposure reversing MDR phenotype were investigated both in vitro and in vivo.

**Results:**

Combination of ADM and 0.74 W/cm^2^ US irradiation enhanced ADM intracellular concentration and nuclear accumulation in MCF-7/ADR and HEPG2/ADM cells, compared to those treated with ADM alone. Enhanced cellular ADM uptake and nuclei localization was associated with increased cytotoxicity of ADM to ADM-resistant cells, lower ADM-resistant cell viability and proliferative cell ratio, and higher apoptotic cell ratio. More importantly, US exposure increased the effectiveness of ADM to inhibit tumor growth in MCF-7/ADR xenograft mice. Mechanistically, US exposure promoted ADM accumulation in MDR cells mainly through down-regulation of P-glycoprotein (P-gp), which is dependent on US-induced intracellular reactive oxygen species (ROS) production. US-induced oxidative stress promoted miR-200c-3p and miR-34a-3p expression by forming miR-200c/34a/ZEB1 double-negative feedback loop. Finally, US-induced miR-200c/34a overexpression decreased P-gp expression and reversed MDR phenotype.

**Conclusion:**

US irradiation could reverse MDR phenotype by activating ROS-ZEB1-miR200c/34a-P-gp signal pathway. Our findings offer a new and promising strategy for sensitizing cells to combat MDR and to improve the therapeutic index of chemotherapy.

**Electronic supplementary material:**

The online version of this article (10.1186/s13046-018-0900-6) contains supplementary material, which is available to authorized users.

## Background

Chemotherapy is one of the most effective treatments for malignant tumors. The progressive inducement to multidrug resistance (MDR), however, is often responsible for the final failure of chemotherapy treatment, and it is believed to be one of the leading reasons making cancers incurable [1, 2]. It is estimated that nearly 90% of the cancer patients with metastasis fail in their treatment due to developed MDR [3]. Although multifactorial in mechanism, enhanced drug efflux mediated by membranal P-glycoprotein (P-gp) is believed to be one major cause of cancer MDR [4, 5]. P-gp, also known as ATP binding cassette subfamily B member 1 (ABCB-1) or MDR1, is a member of the ATP-binding cassette transporter family that prevents anticancer drugs from intracellular accumulation to a therapeutic level by extruding these drugs across the cytomembrane [6, 7]. Cancer drugs such as adriamycin (ADM), paclitaxel, daunorubicin, and epirubicin are common substrates of P-gp. Evidence from preclinical models revealed that overexpression of P-gp led to MDR resistance against multiple cancer drugs; in cancer patients, P-gp upregulation was related to suboptimal treatment response and poor long-term prognosis [8]. Therefore, the inhibition of P-gp is one of the most extensively studied strategies for MDR reversal.

Unfortunately, current solutions for P-gp mediated MDR are not sufficiently effective or safe/far from being satisfactory. Although three distinct generations of P-gp inhibitors have been produced in the past 30 years, a clinically serviceable modulator has yet to be developed [9]. A major deficiency of P-gp inhibitors is their indiscriminate distribution among organs and nonspecific action on P-gp [10]. Since P-gp has shown a protective role in several important organs and tissues, such as indigestive system, the blood-testis barrier, the blood-brain barrier, and membranes of many types of stem cells [11], systemic administration of P-gp inhibitors may lead to systemic toxicity. To overcome this toxicity, researchers are investigating the use of a nanoparticle-mediated specific drug delivery system to tumor tissues to restrict irrelevant P-gp inhibition [12]. Recent studies have also explored the efficacy of local prevention of the biosynthesis of P-gp in tumors using RNAi or miRNAs delivered by viral vectors [13–15]. These efforts are similarly hindered, however, by challenges such as accurate viral location, expression efficiency, stability, and administration safety in vivo. Taken together, these applications are still problematic in their translation from experiments to clinic.

Ultrasound (US), a form of mechanical energy, has been shown the ability to open cell membranes, enhancing delivery of drugs, proteins, and genes through what is known as sonoporation effect [16]. Intriguingly, recent studies have revealed that US exposure significantly repressed P-gp expression [17–21], suggesting that US exposure may facilitate intracellular accumulation of chemotherapy drugs in MDR cancer cells. Because the mechanical energy of US can be focused on an area as tiny as at millimeter level, US exposure could accurately restrict the sonoporation effect or P-gp inhibition to an appointed target, thus avoiding toxicity in nontarget organs. Therefore, US exposure may be a promising approach in the treatment of MDR for its ability to locally increase anticancer agent concentrations in MDR cancer tissues. However, the efficacy and feasibility of this new approach in sensitizing drug-resistant cells to chemotherapy treatment and improving long-term outcomes has not been evaluated in vivo. Moreover, the underlying mechanism by which US decreased P-gp expression is still largely unknown.

In the current study, our observations demonstrated that US exposure increased cytotoxicity of ADM to ADM-resistant cells both in vitro and in vivo. Further, we linked the anti-MDR effect of US exposure to down-regulation of P-gp. US exposure promoted reactive oxygen species (ROS) generation and triggered a double-negative feedback loop involving ZEB1 and miR-*200c*/34a. US-induced miR-200c/34a directly or indirectly inhibited P-gp expression and reversed MDR phenotype.

## Methods

### Cell culture

We purchased human umbilical vein endothelial cell (HUVEC) lines, MCF-7, HEPG2, MCF-7/ADR, and HEPG2/ADM cell lines from Geneseed (Guangzhou, China). The cells were cultured in RPMI 1640 (Gibco, USA) containing 10% fetal bovine serum (FBS; Gibco Laboratories, Gaithersburg, MD, USA), 100 U/ml penicillin and 100 μg/ml streptomycin (Gibco), and 2 mM L-glutamine. To maintain the drug-resistant phenotype, we cultured MCF-7/ADR and HEPG2/ADM cells in the presence of 1 μg/ml ADM (MedChemExpress, Monmouth Junction, NJ, USA) and passaged the cells for 1 week in a drug-free medium before beginning the experiment. We maintained the cells in a humidified incubator in the presence of 5% CO2.

### ADM uptake and intracellular distribution in cells

To detect ADM accumulation in cells, HUVEC, MCF-7, HEPG2, MCF-7/ADR or HEPG2/ADM cells were at 2 × 10^6 cells/well in 6-well plates and incubated overnight. Unless otherwise stated, cells were immediately subjected to US irradiation after addition of ADM. After that, cells were incubated for another 24 h. Then, we rinsed the cells to remove un-internalized ADM. To quantitatively determine cellular ADM concentrations, the treated cells were collected and lysed with RIPA cell lysis buffer (BestBio, Shanghai, China), and the ADM concentration in the cell lysates was detected using a microplate reader (Synergy™ 4, BioTek Instruments, Winooski, VT, USA) at excitation and emission wavelengths of 485/550 nm [22]. We normalized the results to total cellular protein content, which we determined using the BCA protein assay kit (BestBio, China).

To determine the intracellular distribution of ADM, the cells were seeded in a confocal culture dish at a concentration of 5X10^5 cells. Following treatment, we rinsed the cells three times with PBS and fixed them with a 4% paraformaldehyde solution for 30 min at room temperature. We then rinsed the cells with PBS three times for 5 min each time, and stained the cells with DAPI for 5 min. We then washed the cells three times with PBS to remove extracellular DAPI and examined the cells with a confocal laser scanning microscope (LSM 880 with Airyscan, Carl Zeiss, Germany) to view localized ADM in the cells at excitation and emission wave lengths of 485/550 nm.

### Cytotoxicity assay

We seeded HUVEC, MCF-7, HEPG2, MCF-7/ADR or HEPG2/ADM cells at 10^6^ cells/well in 6-well plates and incubated the cells for 24 h. Cells were incubated with ADM or other modulators. Then, US irradiation was performed as stated above. After 24 h treatment, we determined cell viability via MTT assay as described previously [23]. We measured absorbance at 450 nm using a multimode plate reader (Synergy™ 4, BioTek Instruments). Bliss model was used to calculate the IC50 of ADM as described previously [24].

### Determination of optimal US parameters in vitro

A pulsed therapeutic ultrasound device with a KHT-017 transducer (DCT-700, Shenzhen Well.D Medical Electronic, Shenzhen, China) was used for ultrasound stimulation. As described previously [25], the sterilized transducer was fixed on a steel stand with scale to maintain a distance of 10 mm between the transducer and cultured cell in monolayers (Additional file 1: Figure S1). The transducer was operated at a frequency of 1 MHz with a pulse repetition frequency of 10 Hz and a duty cycle of 20% for 5 min.

To determine the optimal acoustic intensity of US in vitro, we seeded MCF-7/ADR at a concentration of 10^6 cells in 6-well plates and incubated the cells for 24 h to allow for adhesion. ADM was added to medium before application of US under differentiated peak negative acoustic intensities (0, 0.09, 0.21, 0.40, 0.74, and 1.22 W/cm^2^). Then cells were incubated for another 24 h. We detected cell viability via MTT assay. We detected ADM fluorescence and intracellular distribution as described above.

### Flow cytometry analysis

MCF-7/ADR or HEPG2/ADM cells were treated with ADM or ADM + US. At 24 h after treatment, we determined cell apoptosis using flow cytometry (LSRFortessa™, BD Biosciences, San Jose, CA, USA) via PI and FITC (BestBio) staining. Cells were collected and washed three times in ice-cold PBS. Cells were resuspended in 400 μl of Annexin V which contained 5 μl Annexin V-FITC. Following 15 min of incubation at 4 °C, we added 10 μl of PI to the suspension and incubated for 5 min. We detected apoptosis using flow cytometry (BD LSRFortessa X-20).

### Scanning Electron microscopy

We observed structural changes in cells following exposure to the optimum US conditions (immediately, 30 min, 60 min, 24 h), as compared with cells without US irradiation, using SEM. We seeded HUVEC, MCF-7, HEPG2, MCF-7/ADR or HEPG2/ADM cells at a concentration of 10^6 cells in 6-well plates and incubated the cells for 24 h to allow for adhesion. We performed US with the determined optimal parameters as described above. Following treatment, we washed the cells twice with PBS and resuspended the cells in PBS. Cell suspensions were dropped on a cover glass with a gold-plated membrane for 30 min. The cells were fixed with 4% formalin for 2–3 min and then were washed with triple PBS for 10 min. The cells were then fixed with 1% osmic acid for 20–30 min and then were washed with ice-cold distilled water three times. Then the cells were soaked in 2% tannin at 4 °C overnight, dehydrated using graded ethanol, and lyophilized using tertiary butyl alcohol overnight. Finally, the cells were coated using a vacuum spray-plating instrument (Hitachi H-7500) and images were captured.

### Preparation of MCF-7/ADR xenograft nude mice

We purchased 60 female BALB/C-nude mice aged 4 weeks from Guangdong Medical Laboratory Animal Center (GDMLAC). Following subcutaneous injection of 10^7 MCF-7/ADR cells into the right oxter, we observed nude mice for 4 weeks, or until solid tumor growth reached 0.5–1.0 cm^3^. We calculated tumor volume (V) as follows: π × (L × W^2^)/6 (L: length, W: width) [26].

### Determination of optimal US parameters in vivo

To explore an appropriate level of ultrasound intensity, 24 MCF-7/ADR xenograft nude mice were randomized into six group treated with ADM (8 mg/kg) intravenously (i.v.) + US exposure of differentiated peak negative acoustic intensities (0.09, 0.21, 0.40, 0.74, and 1.22 W/cm^2^) every 2 days (q2d). The US transducer was placed in contact with skin overlying the xenograft. The other parameters of US stimulation in vivo were the same as that used in vitro. After 7 days, the mice were sacrificed. The tumor tissues and surrounding muscle tissues were isolated for further experiments. Tissue apoptosis was detected by terminal deoxynucleotidyl transferase dUTP nick end labeling (TUNEL) staining per the manufacturer’s protocol (Roche-Diagnostics, Indianapolis, IN, USA). We detected the apoptotic cells (stained red) using confocal microscopy, ADM fluorescence of tumor tissues, and surrounding muscle tissue using microplate readers. The intensity of US exposure, which enhanced intracellular ADM concentrations without inducing apoptosis in surrounding muscle tissues, were used for further studies.

### Reversal of MDR in vivo

To assess the synergistical effect of US exposure and ADM treatment in vivo, 12 MCF-7/ADR xenograft nude mice were randomly subjected into treatment groups of three, including groups receiving ADM alone (8 mg/kg, i.v. q2d), both ADM (8 mg/kg, i.v.) + US exposure (2 min), q3d. The drug dosage was modulated according to the animal’s weight every 6 days. Tumor growth was monitored since the first day of treatment and the tumor volume was measured every 6 days. Twenty-four days later, the mice were sacrificed. The excised tumor tissues were used for further experiments.

### Immunohistochemical staining

Tumor tissues were fixed with 4% paraformaldehyde and paraffin-embedded. De-paraffinized sections were rehydrated through a graded alcohol series. Sections were boiled in antigen retrieval solution (10 mM sodium citrate, 0.05% Tween-20, pH 6.0) for 20 min and then cooled for 30 min. After blocking with 4% normal goat serum, the sections were incubated overnight with P-glycoprotein mAb (ab170904, Abcam), ZEB1 mAb (21544–1-AP, ProteinTech). The expression of P-gp or ZEB1 was assessed as described previously [27].

### Immunofluorescence staining

Tumor tissues were collected and embedded in tissue-freezing medium. Cultured cells were fixed in 4% PFA (10 min). Then tissue sections and cultured cells were processed in the following methods. Samples were blocked with 4% normal goat serum and incubated for 3 h at room temperature with primary antibodies. The antibodies used are followed: anti-Ki67 antibody (1:200, 275R, Cell Marque), anti-P-gp antibody (ab170904, Abcam). When indicated, cells were stained using Click-it EdU imaging Kit (Life Technologies, #C10638) to detect EdU incorporation, according to the manufacturer’s instructions. Then, samples were washed with PBS and stained with secondary antibodies (Alexa Fluor 647, Abcam) for 1 h at room temperature. DAPI was used for the nuclear visualization. Image acquisition was performed using the confocal microscope (Carl Zeiss).

### ADM uptake and distribution in tumor tissue

To detected ADM uptake in tissues, tissues were excised and lysed in lysis buffer after mice were sacrificed. After incubation for 30 min at room temperature and centrifugation at 14,000 rpm for 10 min, supernatant was transferred to a 96-well plate. ADM concentration in the supernatant was detected using microplate readers at excitation and emission wavelengths of 485/550 nm. The results were normalized to total tissue protein content.

To detect ADM distribution in tissues, twenty-four hours after ADM administration, mice were sacrificed and tissues were collected and embedded in tissue-freezing medium. Tumor tissues were sectioned (10 μm thick) and imaged using a confocal microscope.

### TUNEL staining

Tumor tissues were fixed with 4% paraformaldehyde and paraffin-embedded. Cultured cells were fixed in 4% PFA (10 min). Then tissue sections and cultured cells were processed using In Situ Cell Death Detection Kit (Roche, Shanghai, China) to stain the apoptotic cells. Nuclei were stained with DAPI. We observed the tissue or cells using inverted fluorescence microscope (Olympus, Hamburg, Germany).

### Real-time polymerase chain reaction

We isolated total RNA from cells or tissues by Trizol (Takara Bio, Inc., Shiga, Japan).β-catenin and U6 genes were used as gene and miRNA internal controls, respectively. Quantitative Polymerase chain reaction (Q-PCR) was performed by using the SYBR Green PCR Master Mix (TOYOBO Corp., Osaka-fu, Japan) in LightCycle480 (Roche, Germany). Cyclin conditions were as following: initial denaturation at 95 °C for 10 min, followed by 40 cycles of 95 °C for 15 s, 60 °C for 15 s, 72 °C for 32 s. Specific sense primers for ABCB1, miR-200c, miR-34a-3p, U6, ZEB1 and β-actin are shown in Additional file 2: Table S1.

### Western blot analysis

We extracted total protein from treated cells or tissues using RIPA lysis buffer (BestBio) containing protease inhibitor cocktail Set I (BestBio). We separated proteins in 10% precast SDS-PAGE gels and transferred them onto a nitrocellulose membrane. The membrane was further incubated with primary P-glycoprotein mAb (1:100 dilution; ab170904, Abcam), ZEB1 mAb (1:100 dilution; 21544–1-AP, ProteinTech) or β-actin mAb (1:1000 dilution; Santa Cruz, CA, USA). We visualized the blots using an odyssey detection system (LI-COR Biosciences, Lincoln, NE, USA) and quantified protein abundance by ImageJ software.

### Reactive oxygen species detection

We detected the intracellular generation of ROS in cells using 2′,7′-dichlorofluorescein-diacetate (DCFH-DA; Beyotime, Jiangsu, China). We seeded MCF-7/ADR or HEPG2/ADM cells at a concentration of 10^6 cells in 6-well plates and incubated the cells for 24 h to allow for adhesion. We added DCFH-DA at a final concentration of 10 μM at 24 h post-treatment and incubated for another 20 min at 37 °C. Cells were then lysed, and the lysates were centrifuged at 10,000×g at 4 °C for 5 min. We transferred the supernatant to black 96-well plates and measured the supernatant using a microplate reader at an excitation wavelength of 488 nm and emission wavelength of 525 nm. Relative fluorescence units of the samples were calculated and normalized to the untreated cells. We also observed the cells by inverted fluorescence microscope (Olympus, Hamburg, Germany) after they were incubated for 20 min at 37 °C in 10 μM DCFH-DA.

### MiRNA mimics, miRNA inhibitors or siRNA transfection

MCF-7/ADR cells (1*10^7^ count) were seeded in 6-wells plates on the day before the transfection. MiR-200c mimic (100 pmol, RIBOBIO), miR-34a-3p mimic (100 pmol, RIBOBIO), mimic negative control (mimic-NC, 100 pmol, RIBOBIO), anta-miR-200c (100 pmol, RIBOBIO), anta-miR-34a-3p (100 pmol, RIBOBIO), antagomir for negative control (anta-NC, 100 pmol, RIBOBIO), siRNA for ZEB1 (20 nM, RIBOMO) or siRNA for negative control (si-NC, 20 nM, RIBOMO) was used for the transfection of the cells, which was achieved by using Lipofectamine 2000 transfection reagent (Invirogen) according to manufacturers’ protocol. At 24 after transfection, cells were treated with ADM or ADM + US as stated above.

### Luciferase assay

The ZEB1 3′-UTR or ABCB1 3′-UTR sequence was PCR-amplified and cloned into the luciferase vector psiCHECK-2 (Saicheng Bio Co Ltd., China). The used primers for PCR amplification are shown in Additional file 2: Table S1. MCF-7/ADR cells were seeded in 24-well plates 24 h prior to transfection. For luciferase report assays, the miR-34a-3p or miR-200c mimic was co-transfected into MCF-7/ADR cells with constructed luciferase vectors. Firefly and Renilla luciferase activity was detected by the Dual Luciferase Reporter Assay system (Promega, Madison, WI).

### Chromatin immunoprecipitation assay

The EpiQuik chromatin immunoprecipitation assay kit (EpiGentek, Brooklyn, NY) was applied according to the manufacturer’s instructions. A 5 μg portion of control IgG antibody or anti-ZEB1 antibody was used for immunoprecipitation. PCR was used to investigate enrichment of DNA fragments in the predicted ZEB1 binding sites in the miR-200c/34a promoters. The used primers are shown in Additional file 2: Table S1.

### Statistical analysis

We displayed data as means plus or minus standard deviations. We calculated mean values from at least three experiments. We performed multiple comparisons using one-way analysis of variance (ANOVA) followed by Bonferroni correction. Differences were considered significant at *P < 0.05. We performed statistical analyses using SPSS 13.0 (IBM SPSS, Chicago, IL, USA).

## Results

### US exposure enhances ADM-inducing cell killing in vitro

**Fig. 1:**
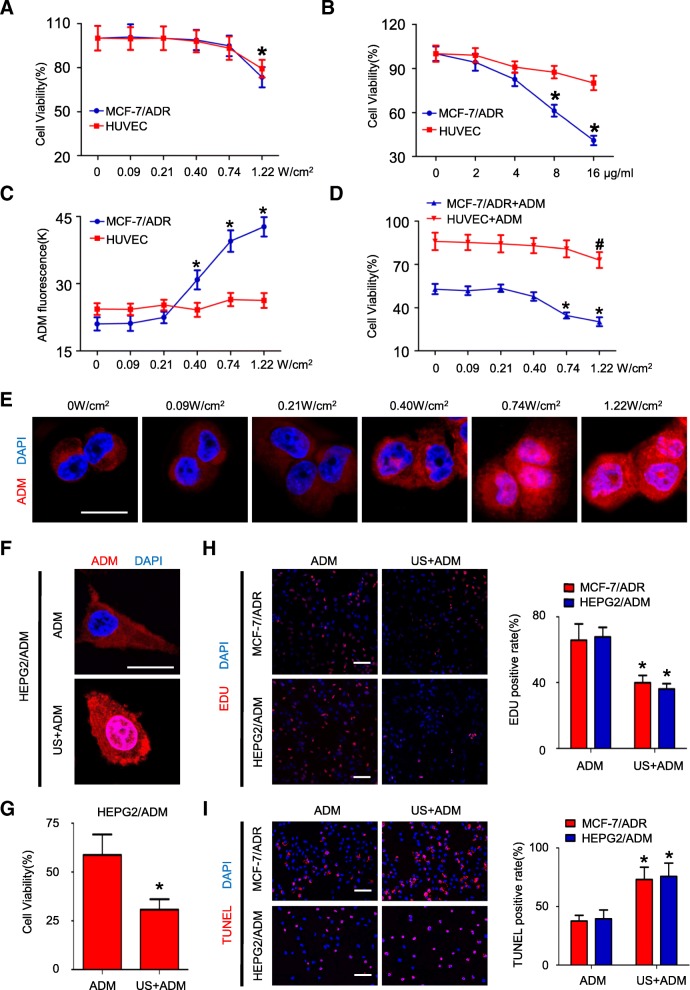
Exploration of optimal US parameters for reversing MDR in vitro. **a** Cell viability in MCF-7/ADR and HUVEC cells in 24 h after US exposure with different acoustic intensities; *N* = 3; *P < 0.05 vs. 0 W/cm^2^ in MCF-7/ADR cells; (**b**) Cytotoxicity of ADM alone in the MCF-7/ADR and HUVEC cells; N = 3, *P < 0.05 compared with HUVEC cells; (**c**) Intracellular ADM concentration in MCF-7/ADR and HUVEC cells in 24 h after US exposure with different acoustic intensities; N = 3; *P < 0.05 vs. 0 W/cm^2^ in MCF-7/ADR cells; (**d**) Cytotoxicity of ADM in MCF-7/ADR and HUVEC cells post US+ADM treatment with different acoustic intensities; N = 3; *P < 0.05 vs. 0 W/cm^2^ in MCF-7/ADR cells; data are represented as mean ± s.d; (**e**) Images of intracellular ADM distribution in MCF-7/ADR cells post US+ADM treatment with different acoustic intensities (scale bar = 10 μm); (**f**) Ultrasound acoustic intensity of 0.74 W/cm^2^ enhanced intracellular ADM uptake and ADM nuclei localization in HEPG2/ADM cells; (**g**) Cytotoxicity of US+ADM or ADM alone in HEPG2/ADM cells; N = 3; data are represented as mean ± s.d; *P < 0.05; (**h**) EDU staining and quantification of the proliferative cells in MCF-7/ADR and HEPG2/ADM cells post-treatment with US+ADM or ADM alone (scale bar = 50 μm); N = 3; data are represented as mean ± s.d; *P < 0.05; (**i**) TUNEL staining and quantification of the apoptotic cells in MCF-7/ADR and HEPG2/ADM cells post-treatment with US+ADM or ADM alone (scale bar = 50 μm); N = 3; data are represented as mean ± s.d; *P < 0.05

To determine the optimal US parameters which increase cytotoxicity of chemotherapy drugs for MDR cells but not for healthy cells, we observed the effect of ultrasound exposure on both MCF-7/ADR and HUVEC cells. We first studied whether US exposure itself inhibits cell growth in the MCF-7/ADR and HUVEC cells. Cell viability was more than 90% in both the MCF-7/ADR cells and HUVEC cells using ultrasound acoustic intensity ≤0.74 W/cm^2^ exposure (Fig. 1a). Then, we examined MCF-7/ADR cells and HUVEC cells for their response to ADM treatment. The IC50 of ADM concentration for MCF-7/ADR cells is 12.19 ± 1.65 μg/ml (Additional file 3: Figure S2A and Additional file 4: Table S2). MCF-7/ADR cells are more sensitive to ADM compared with HUVEC cells (*P* < 0.05, ≥8 μg/ml ADM; Fig. 1b). Their different response to ADM treatment might result from higher mitotic rate of cancer cells. The half-inhibitory concentration (IC50) dosage of ADM for MCF-ADR cells was used in the following experiments in vitro. Next, MCF-7/ADR cells and HUVEC cells were incubated with ADM and treated with ultrasound of different acoustic intensities. We assessed ADM accumulation and retention following US exposure. Expectedly, intracellular ADM concentrations were significantly enhanced in MCF-7/ADR cells with the increase in acoustic intensity (*P* < 0.05, ≥0.40 W/cm^2^; Fig. 1c). Moreover, ultrasound acoustic intensity of 0.74, 1.22 W/cm^2^ increased the quantity of ADM nuclei localization in MCF-7/ADR cells (Fig. 1e). We further explored whether US exposure could elevate the cytotoxicity of ADM against ADM-resistant cells. The viability of MCF-7/ADR cells with US+ADM treatment decreased significantly with the increase in ultrasound acoustic intensity (*P* < 0.05, ≥0.74 W/cm^2^, Fig. 1d). In contrast, intracellular ADM concentrations in HUVEC did not increased after US exposure (Fig. 1c). The cell viability in HUVEC was slightly reduced after treated with ADM + 1.22 W/cm^2^ US exposure (*P* < 0.05, vs. 0 W/cm^2^; Fig. 1d), which might result from US itself cytotoxicity. Considering 0.74 W/cm^2^ US largely enhanced sensitivity to ADM of ADM-resistant cells but had minor effect on HUVEC, 0.74 W/cm^2^ was selected as the optimal acoustic intensity in vitro.

In order to evaluate whether US exposure reversed MDR in other drug resistant cells, HEPG2/ADM cell line, another MDR cell line, was also utilized in the following experiments. The IC50 dosage of ADM for HEPG2/ADM cells was 10.26 ± 1.29 μg/ml. The IC50 dosage of ADM was used in the following studies involving HEPG2/ADM cells (Additional file 3: Figure S2B and Additional file 4: Table S2). Ultrasound acoustic intensity of 0.74 W/cm^2^ increased the quantity of ADM nuclei localization in HEPG2/ADM cells (Fig. 1f). Furthermore, the viability of HEPG2/ADM cells treated with US+ADM was significantly lower than that of cell treated with ADM alone (29.15 ± 2.08% vs. 53.94 ± 3.16%; *P* < 0.05, Fig. 1g). The IC50 of ADM concentration in US+ADM treatment decreased 40% and 38% compared with ADM group in MCF-7/ADR and HEPG2/ADM cells, respectively (*P* < 0.05; Additional file 4: Table S2 and Additional file 3: Figure S2A and B).

The decreased cell viability in US+ADM group is likely attributed to increased sensitivity to ADM-induced proliferation inhibition and apoptosis. We detected the proliferation profile of MCF-7/ADR and HEPG2/ADM cells using EDU staining. As shown in Fig. 1h, the treatment of US+ADM showed a decreased proliferative cell population in MCF-7/ADR and HEPG2/ADM cells, compared to ADM treatment (42.81 ± 4.83%, 39.76 ± 4.07% vs 65.70 ± 4.36%, 67.49 ± 4.69%; *P* < 0.05; respectively). Additionally, TUNEL assays and flow cytometry assays were performed to determine the apoptotic index of these cells. For TUNEL staining, the percentage of MCF-7/ADR and HEPG2/ADM cells with apoptotic features induced by ADM treatment was 37.6 ± 4.92% and 39.45 ± 7.60%, respectively. When combined with US exposure, it increased to 73.19 ± 9.82% and 75.72 ± 9.01%, respectively (*P* < 0.01, compared with ADM group, Fig. 1i). Similar to these results, flow cytometry assays showed that apoptotic cell ratio at G2 + G4 stage of MCF-7/ADR and HEPG2/ADM cells treated with ADM alone was 22.70 ± 4.92% and 24.7 ± 6.08% of cells, whereas 80.91 ± 7.41% and 86.10 ± 6.47% of cells treated with US+ADM, respectively (*P* < 0.01, compared with ADM group, Additional file 3: Figure S2C).

### Sonoporation effect of US exposure briefly enhances the accumulation and retention of ADM in MDR cells

**Fig. 2:**
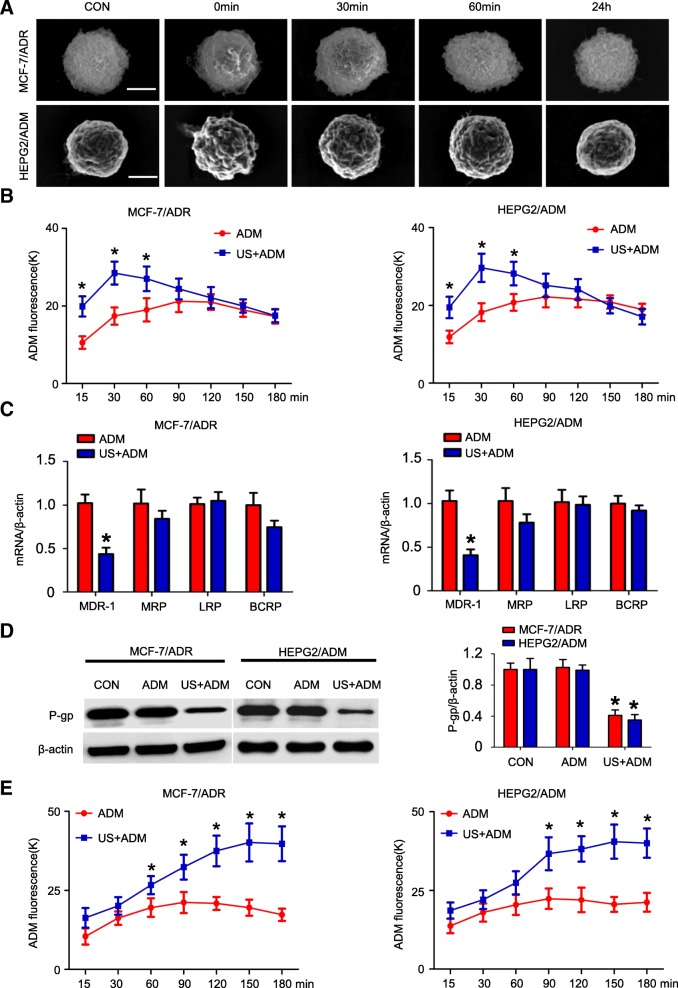
The mechanisms of US-mediated ADM accumulation. **a** Cell morphology and cytomembrane changes after US exposure (scale bar = 5 μm); (**b**) The dynamic change of ADM concentration in MCF-7/ADR and HEPG2/ADM cells treated with ADM immediately after US exposure; N = 3; data are represented as mean ± s.d; *P < 0.05; (**c**) Q-PCR of drug efflux transporters mRNA in MCF-7/ADR and HEPG2/ADM cells in 24 h after US exposure or not; N = 3; data are represented as mean ± s.d; *P < 0.05; (**d**) Immunoblotting of P-gp expression in 24 h after US exposure; N = 3; data are represented as mean ± s.d; *P < 0.05; (**e**) The dynamic change of ADM concentration in MCF-7/ADR and HEPG2/ADM cells treated with US+ADM or ADM alone. ADM was added to the mediums in 24 h after exposure to US; N = 3; data are represented as mean ± s.d; *P < 0.05

We studied the potential mechanisms by which US exposure reversing MDR. We firstly explored whether ADM accumulation in response to US exposure is mediated by sonoporation effect which often disrupts the cell membrane. As is shown in Fig. 2a, cell membranes became crimpled and micropores development was observed immediately after exposure to US. However, the micropores disappeared and cytomembranes recovered normal shape at 24 h post-exposure. To investigate the role of US-induced sonoporation in reversal of MDR, we added ADM to culture dishes at 5 min after exposure to either US exposure or control treatment and monitoring intracellular ADM concentration. ADM concentrations peaked in 30 min post-treatment in US+ADM group and 90 min post-treatment in ADM group (Fig. 2b). The peak of ADM concentrations in US+ADM group were higher than that of ADM groups in MCF-7/ADR and HEPG2/ADM cells by more than 50% (*P* < 0.05; respectively). In 120 min post-treatment in US+ADM group, ADM concentrations decreased to the comparable levels as the ADM group in both cell lines (*P* > 0.05; Fig. 2b). These findings indicated sonoporation might mediate short-term effect of US exposure in enhancing ADM accumulation.

### Down-regulation of P-gp is mainly attributed to US-mediated MDR reversal

US exposure has been reported to decrease drug transporter expression. We, therefore, explored whether US exposure reversed MDR through repressing drug efflux transporter expression. We detected the expression level of MDR-1, MRP, LRP and BCRP gene 24 h after treatment. P-gp (MDR-1) mRNA expression level in cells treated by US+ADM decreased more obviously than others (Fig. 2c). In agreement with these results, immunoblotting showed that both P-gp protein expression was significantly decreased in 24 h after US exposure in MCF-7/ADR and HEPG2/ADM cells (*P* < 0.05; respectively; Fig. 2d). To determine the role of P-gp down-regulation in US-mediated MDR reversal, we added ADM to culture dishes 24 h after cells were stimulated by US or control treatment. In US+ADM group, ADM concentrations showed a time-dependent increase and reached a plateau at 120 min post treatment in MCF-7/ADR cells and at 90 min in HEPG2/ADM cells (Fig. 2e). The peak of ADM concentrations in both US+ADM group were more than twice as much as that of ADM group in MCF-7/ADR and HEPG2/ADM cells (*P* < 0.05; respectively). Taken together, these findings demonstrated a critical role of P-gp down-regulation in US-induced MDR reversal.

### ROS activation is responsible for US exposure mediated P-gp down-regulation

**Fig. 3:**
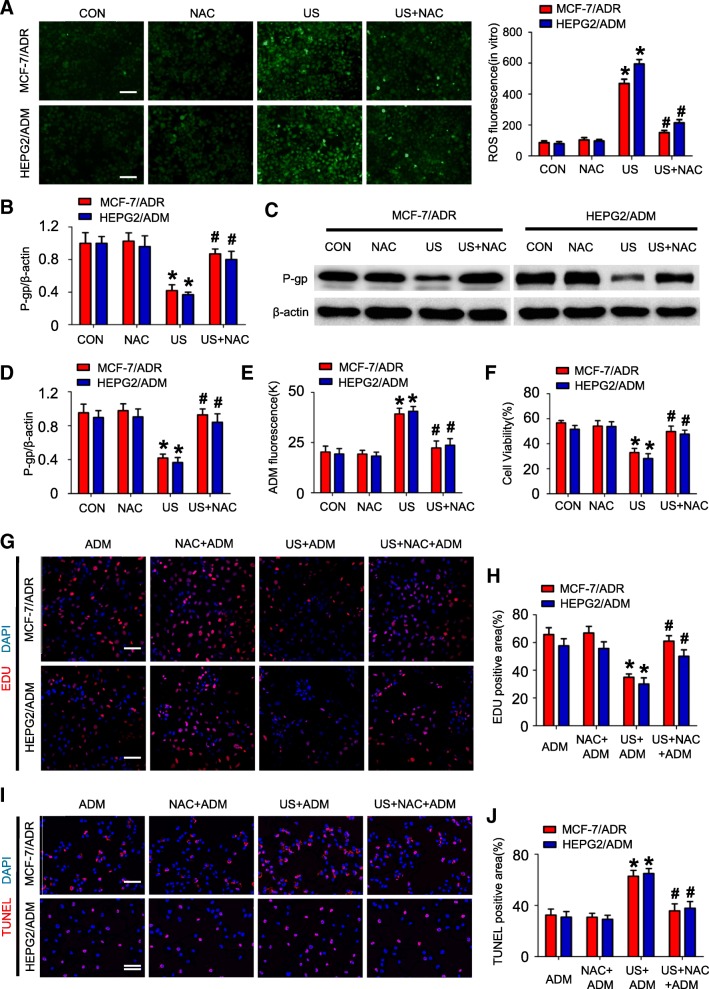
US exposure induces P-gp down-regulation is dependent on intracellular ROS generation. **a** US induced ROS generation in MCF-7/ADR and HEPG2/ADM cells which could be blocked by pre-treated with 5 mM NAC for 2 h (scale bar = 50 μm); Representative images of DCFH-DA staining in MCF-7/ADR and HEPG2/ADM cells 24 h post-treatment; N = 3; data are represented as mean ± s.d; *P < 0.05 compared with CON group; (**b**-**d**) Pre-treated with 5 mM NAC for 2 h inhibited US-induced P-gp down-regulation; Q-PCR assays (**b**), Western blot assays (**c**-**d**); (**e**-**j**) MCF-7/ADR and HEPG2/ADM cells were pre-treated with or without 5 mM NAC for 2 h before exposure to US+ADM. ADM intracellular concentration was determined by measuring fluorescent intensity (**e**); Cell viability was determined by MTT assay (**f**); Percentage of proliferative cell was determined by EDU staining (**g**-**h**, scale bar = 50 μm); Percentage of cell apoptosis was determined by TUNEL staining (**i**-**j**, scale bar = 50 μm); *N* = 3; data are represented as mean ± s.d; *P < 0.05 compared with ADM group

Previous studies documented that US exposure modulated gene expression via the generation of ROS. We hypothesized US exposure reversed MDR via ROS mediated transcriptional repression of P-gp. As is shown in Additional file 5: Figure S3, US exposure significantly increased ROS activity in MCF-7/ADR and HEPG2/ADM cells with the time increase (≥ 4 h, *P* < 0.05, respectively). To determine if elevated ROS contributed to P-gp down-regulation, a ROS scavenger N-acetyl-L-cysteine (NAC) was used. It was showed that pre-treated with 5 mM NAC could prevent US-mediated ROS activation (*P* > 0.05; Fig. 3a). Moreover, Q-RTPCR assays and western blot assays showed that US-induced P-gp repression could be attenuated by NAC pre-treatment (US+NAC vs. US, *P* < 0.05; Fig. 3b, c and d, respectively). In addition, pre-treated with 5 mM NAC significantly decreased intracellular ADM concentration (US+NAC + ADM vs. US+ADM, *P* < 0.05; Fig. 3e), apoptotic cell ratio (US+NAC + ADM vs. US+ADM, *P* < 0.05; Fig. 3i and j) and increased cell viability and proliferative cell ratio (US+NAC + ADM vs. US+ADM, *P* < 0.05, respectively; Fig. 3f, g and h) in MCF-7/ADR and HEPG2/ADM cells receiving US+ADM treatment.

### US exposure increases miR-200c/34a expression via oxidative stress

**Fig. 4:**
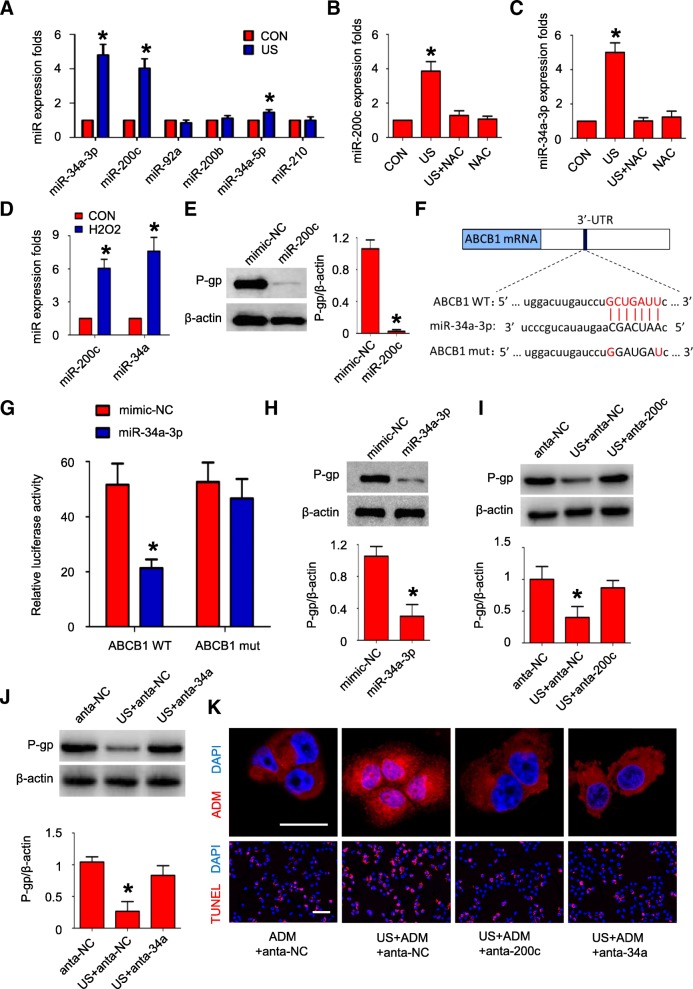
miR-200c/34a participate in US decreasing P-gp expression. **a** Q-PCR of oxidative stress responsive miRNAs in MCF-7/ADR cells in 24 h after US exposure; N = 3; data are represented as mean ± s.d; *P < 0.05; (**b**-**c**) Pre-treated with 5 mM NAC abrogated US-induced miR-200c (**b**) or miR-34a-3p (**c**) overexpression in MCF-7/ADR cells; *N* = 4; data are represented as mean ± s.d; *P < 0.05 compared with CON group; (**d**) MiR-200c/34a expression in MCF-7/ADR cells treated with 400 mM H_2_O_2_; N = 3; data are represented as mean ± s.d; *P < 0.05 compared with CON group; (**e**) P-gp expression in MCF-7/ADR cells transfected with miR-200c mimic; N = 3; data are represented as mean ± s.d; *P < 0.05; (**f**) Predicted miR-34a-3p seed sequence match to the sequence in the 3’ UTR of P-gp (ABCB1) mRNA; (**g**) Verification of ABCB1 as a target gene of miR-34a-3p by the dual luciferase reporter assay; (**h**) P-gp expression in MCF-7/ADR cells transfected with miR-200c mimic; N = 3; data are represented as mean ± s.d; *P < 0.05; (**i**-**j**) MiR-200c (**i**) or miR-34a-3p (**j**) inhibition attenuated US-induced P-gp up-regulation in MCF-7/ADR cells; N = 3; data are represented as mean ± s.d; *P < 0.05 compared with anta-NC group; (**k**) Images of intracellular ADM distribution (Upper, scale bar = 10 μm) and TUNEL staining (lower, scale bar = 50 μm) in MCF-7/ADR cells treated as described in (**i**-**j**); Quantitative analysis of ADM intracellular concentration and TUNEL-positive cell ratio was shown in Fig.S4A and S4B, respectively

ROS often fulfills its biological effect by modulating microRNAs (miRs) expression in cancer [28]. Thus, we hypothesized that oxidative stress-induced miRs serve as a link between US, ROS and P-gp downregulation. We detected the expression level of several oxidative stress-induced miRs that also regulate drug resistance in cancer on 24 h after US exposure. It was shown that miR-200c-3p and miR-34a-3p were markedly overexpressed in both cell lines after US exposure (*P* < 0.01, respectively; Fig. 4a). The effect of US on miR-200c/34a expression could be abrogated by pretreatment with NAC (US+NAC vs. US, *P* < 0.05; Fig. 4b and c). H_2_O_2_-induced miR-200c/34a upregulation further confirmed US exposure elevates miR-200c/34a expression depending on oxidative stress pathway (*P* < 0.05; Fig. 4d). Successively, we assessed miR-200c/34a effect on multidrug resistance. MCF-7/ADR cells was transfected with miR-200c/34a mimics or inhibitors. MiR-200c was shown to repress P-gp–mediated MDR by targeting JNK-2/c-Jun pathway [29]. Consistent with previous studies, we observed that miR-200c significantly decreased P-gp expression in MCF-7/ADR cells (Fig. 4e). Using TargetScan programme, we found a potential miR-34a-3p binding sites on three prime untranslated region (3’ UTR) of P-gp (ABCB1) (Fig. 4f). We further employed luciferase assay system to test whether miR-34a-3p directly binds to 3’ UTR of P-gp. As is shown in Fig. 4g, the luciferase activity was significantly reduced in miR-34a-3p overexpressing cells, whereas the mutant 3’ UTR didn’t reveal a significant response to miR-34a-3p. Expectedly, miR-34a-3p significantly decreased P-gp expression in MCF-7/ADR cells (Fig. 4h). In addition, miR-200c/34a inhibition attenuated US-induced P-gp down-regulation (Fig. 4i and j). The effect of US on enhancing ADM uptake and ADM-inducing apoptosis was diminished in the presence of miR-200c/34a inhibition (Fig. 4k, Additional file 6: Figure S4A and B). Furthermore, both miR-200c and miR-34a mimics enhanced ADM uptake, ADM-induced cytotoxicity, proliferation inhibition and apoptosis in MCF-7/ADR cells (Additional file 6: Figure S4C, D, E and F). These results indicated an important role of oxidative stress-induced miR-200c/34a in US-mediated P-gp downregulation.

### MiR-200c/34a and ZEB1 formed a negative feedback loop

**Fig. 5:**
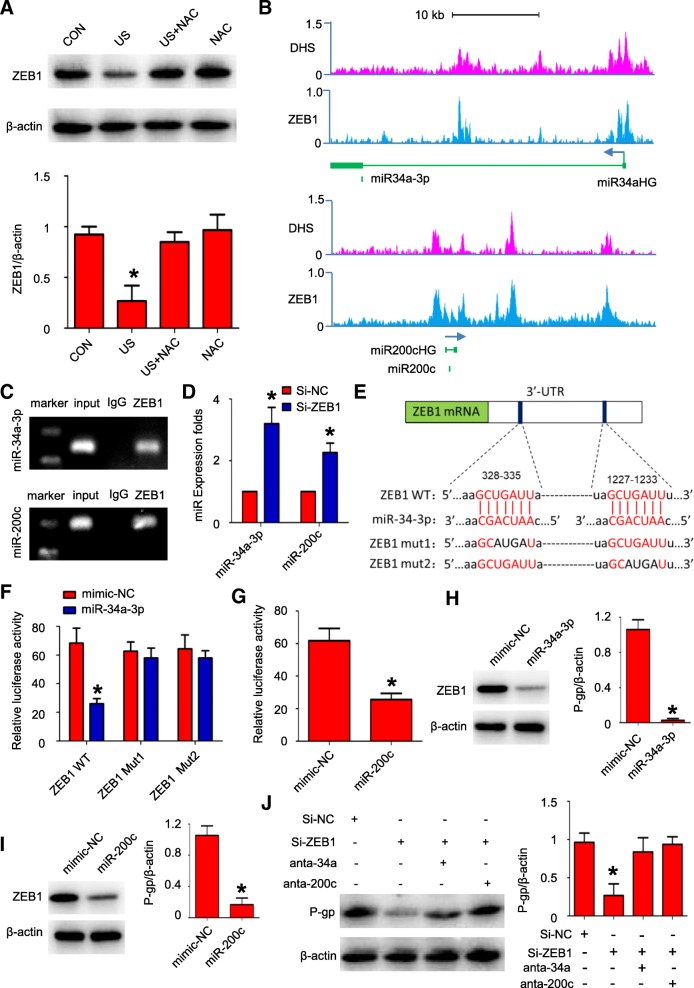
US exposure repressed P-gp expression by activating ZEB1-miR-200c/34a negative feedback loop. **a** US exposure decreased ZEB1 expression in MCF-7/ADR cells, while NAC pretreatment inhibited ZEB1 down-regulation in response to US exposure; N = 3; data are represented as mean ± s.d; *P < 0.05 compared with CON group; (**b**) ZEB1 directly binds to miR-200c/34a promoter in HEPG2 cells. Results were obtained from CHIP-seq of ZEB1 in ENCODE database; (**c**) The ZEB1-binding sites in miR-200c/34a promoter in MCF-7/ADR cells were detected by PCR gel; (**d**) Knockdown of ZEB1 increased miR-200c/34a expression in MCF-7/ADR cells; N = 3; data are represented as mean ± s.d; *P < 0.05; (**e**) Predicted miR-34a-3p seed sequence match to the sequence in the 3’ UTR of ZEB1 mRNA. Mutations were generated in the complementary sequences that match to the seed region of miR-34-3p; (**f**-**g**) Luciferase reporter assay was used to determine miR-34a-3p (**f**) and miR-200c (**g**) direct targeting the ZEB1 3’ UTR; (**h**-**i**) MiR-34a/200c overexpression reduced the ZEB1 expression in MCF-7/ADR cells; N = 3; data are represented as mean ± s.d; *P < 0.05; (**j**) Knockdown of ZEB1 decreased P-gp expression, which could be reserved by miR-34a/200c inhibition; N = 3; data are represented as mean ± s.d; *P < 0.05 compared with si-ZEB1 alone

Previous studies demonstrated the existence of ZEB1/miR-200c double feedback loop is required for oxidative stress induced miR-200c [30]. Thus, we further investigated if US-induced oxidative stress triggers such double feedback loop. We revealed that US exposure decreased ZEB1 expression, which could be prevented by pretreatment of NAC (Fig. 5a). Moreover, chromatin immunoprecipitation followed next-generation sequencing (CHIP-Seq) in HEPG2 cells revealed that ZEB1 occupies regions at the promoters of miR-200c/34a host gene (Fig. 5b). QCHIP assay further confirmed the occupancy by endogenous ZEB1 at miR-200c/34a promoter in MCF-7/ADR cells (Fig. 5c). Accordingly, ZEB1 knockdown elevated miR-200c/34a expression level, indicating ZEB1 repressed miR-200c/34a transcription activity (Fig. 5d). We subsequently explore whether miR200c/34a targets ZEB1. Two potential miR-34a-3p binding sites on 3’ UTR of ZEB1 were found by using TargetScan programme (Fig. 5e). Luciferase assay indicated both binding sites are functional (Fig. 5f). It is also known that miR-200c binds to ZEB1 3’UTR and decreases its expression level [31]. Expectedly, transfection of miR-200c mimic resulted in a significant reduction of luciferase activity of ZEB1 3’UTR in MCF-7/ADR cells (Fig. 5g). Successively, we investigated whether miR-200c/34a affected ZEB1 expression. It turns out to be that miR-200c/34a overexpression reduced ZEB1 expression (Fig. 5h and i). Moreover, knockdown of ZEB1 reduced P-gp expression. MiR-200c/34a inhibition could reverse the effect of ZEB1 knockdown on reducing P-gp expression (Fig. 5j). Taken together, miR-200c/34a and ZEB1 interplays and formed a double negative feedback loop. US triggered this circuitry by activating oxidative pathway, resulting in P-gp down-regulation finally.

### US exposure reverses MDR in vivo

**Fig. 6:**
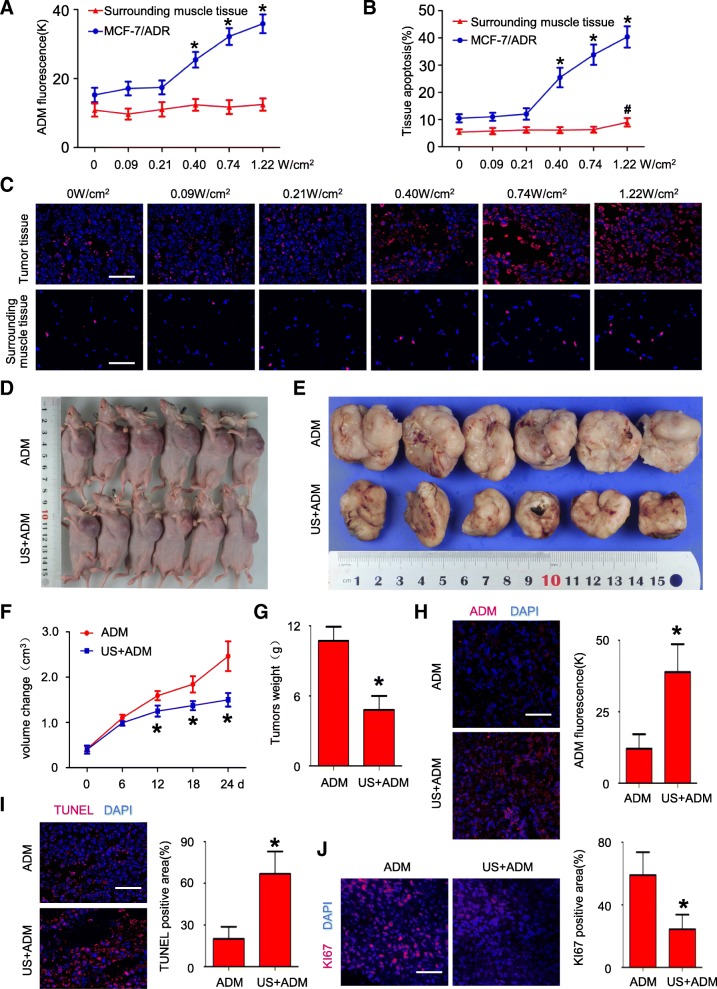
US exposure reversed MDR in vivo. **a** ADM accumulation in MCF-7/ADR xenograft nude mice after US+ADM treatment with different acoustic intensities; N = 4; data are represented as mean ± s.d; *P < 0.05 vs. tumor tissue of 0 W/cm^2^ group; ^#^*P* < 0.05 vs. peritumor muscle tissue of 0 W/cm^2^ group; (**b**-**c**) TUNEL staining in MCF-7/ADR xenograft after treatment with different US acoustic intensities; N = 4; data are represented as mean ± s.d; *P < 0.05 vs. tumor tissue of 0 W/cm^2^ group; ^#^*P* < 0.05 vs. peritumor muscle tissue of 0 W/cm^2^ group; (**d**-**e**) Representative picture of MCF-7/ADR xenograft nude mice (**d**) and isolated tumors (**e**) after US+ADM treatment or ADM treatment on day 24; (**f**) Tumors volumes changes during the treatment; *N* = 6, data are represented as mean ± s.d; *P < 0.05; (**g**) Quantitative analysis of tumors weights after tumors were isolated; *N* = 6, *P < 0.05; (**h**) Tumor uptake of ADM after US+ADM treatment or ADM treatment on day 24; N = 6; data are represented as mean ± s.d; *P < 0.05; (**i**) TUNEL staining on respective tumor tissue to detect apoptotic cells; N = 6; data are represented as mean ± s.d; *P < 0.05; (**j**) Fluorescence signal of Ki-67 staining on respective tumor sections; scale bar = 50 μm; N = 6; data are represented as mean ± s.d; *P < 0.05

To evaluate whether US exposure reverses MDR in vivo, we established MCF-7/ADR xenograft models by subcutaneously inoculating nude mice with MCF-7/ADR cells. We first determined the optimal US Parameters in vivo. MCF-7/ADR xenograft from 24 tumor bearing mice was randomly subjected to ADM (8 mg/kg, i.v.) combined with US irradiation in six different acoustic intensities (0, 0.09, 0.21, 0.40, 0.74, and 1.22 W/cm^2^). We evaluated therapeutic sensitivity of each group by detecting ADM concentration, apoptotic and proliferative index in xenografts one week after treatment. ADM concentrations in MCF-7/ADR tumor tissue were significantly enhanced at 0.40 W/cm^2^, 0.74 W/cm^2^, and 1.22 W/cm^2^ acoustic intensity, compared with 0 W/cm^2^ acoustic intensity (*P* < 0.05, respectively, Fig. 6a). As for peritumoural muscle tissue, ADM concentration was not significantly enhanced with the increase in acoustic intensity (*P* < 0.05, Fig. 6a). Furthermore, the percentage of apoptotic cells were significantly higher in MCF-7/ADR tumor tissue treated with ADM in combination with 0.40 W/cm^2^ (15.41 ± 3.60%), 0.74 W/cm^2^ (28.93 ± 3.77%), and 1.22 W/cm^2^ (34.24 ± 4.94%) US intensity, compared with 0 W/cm^2^ US intensity (10.48 ± 1.49%; *P* < 0.05, respectively), whereas apoptotic cell ratio of peritumoural muscle tissue was significantly higher under 1.22 W/cm^2^ US exposure than 0 W/cm^2^ acoustic intensity (8.98 ± 1.46% vs. 5.41 ± 0.74%; *P* < 0.05; Fig. 6b and c). To avoid the effect of US itself cytotoxicity, 0.74 W/cm^2^ was also determined to be the optimal acoustic intensity in vivo and used in the following in vivo experiments.

We investigated the longer-term beneficial effects of 0.74 W/cm^2^ US exposure on reversal of MDR in vivo. MCF-7/ADR xenograft nude mice were randomly divided into ADM group and US+ADM group. Tumor growth and therapeutic sensitivity were monitored during the course of respective treatment. Xenograft tumor growth curves showed that tumors with ADM treatment alone continued to grow at a steady rate, whereas tumors in US+ADM group grew more slowly (Fig. 6f). Moreover, tumors showed significantly smaller increase of tumor size (Fig. 6d and e) and tumor weight (Fig. 6g) after US+ADM treatment compared with ADM treatment alone (*P* < 0.05, ADM group vs. US+ADM group; respectively). These results indicated that US exposure and ADM treatment impairs MDR tumor growth synergistically in vivo. Moreover, we detected ADM concentration, proliferative and apoptotic index in each group at day 24. ADM concentration in MCF-7/ADR xenograft tissue treated with US+ADM is significantly higher than that in xenograft tissue treated with ADM alone (*P* < 0.05, Fig. 6h). To investigate whether US exposure promotes ADM-mediated apoptosis in MDR tumors, TUNEL staining was used to detected apoptotic tumor cells after 24 days of treatment. As is shown in Fig. 6i, apoptotic tumor cells ratio was significantly higher in US+ADM groups (65.12 ± 7.08%) than ADM groups (12.72 ± 1.09%; *P* < 0.05, respectively). The percentage of ki67 positive cell in US+ADM groups was significantly lower than ADM group (*P* < 0.05, Fig. 6j). Taken together, these in vitro and in vivo experiments demonstrated that US exposure could overcome cancer MDR.

### US exposure decreases P-gp expression via ROS-ZEB1-miR200c/34a pathway in vivo

**Fig. 7:**
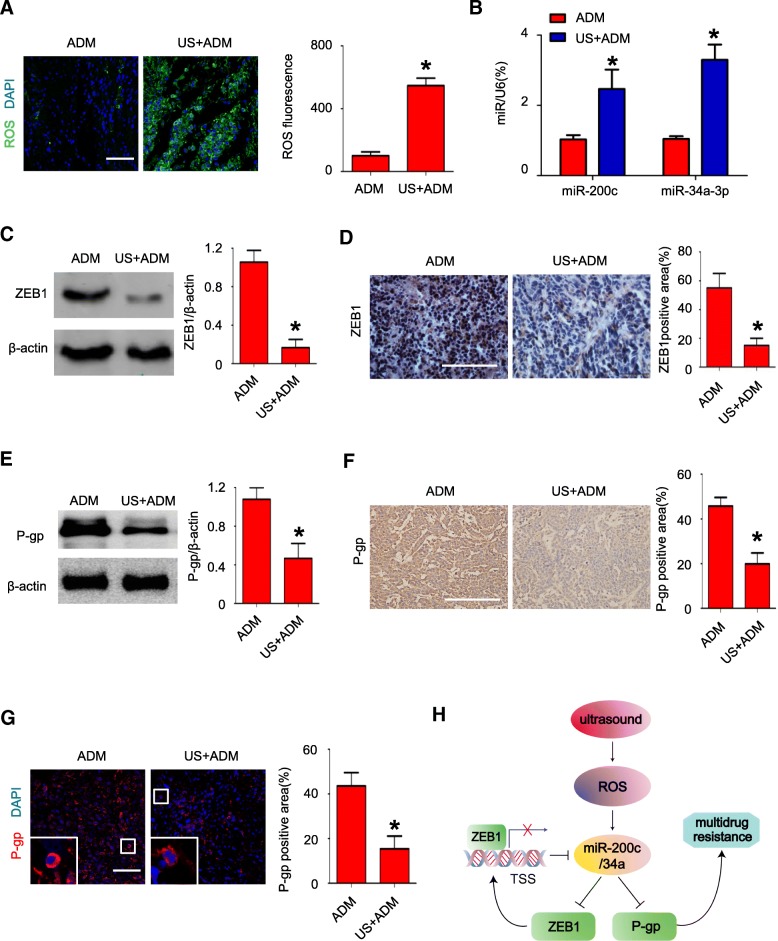
The effect of US exposure on ROS-ZEB1-miR200c/34a-P-gp pathway in vivo. **a** ROS staining (green) of tumor tissue in MCF-7/ADR xenograft nude mice treated with ADM alone or US+ADM; scale bar = 50 μm; N = 6; data are represented as mean ± s.d; *P < 0.05; (**b**) MiR-200c/34a expression levels were quantified by qRT-PCR in MCF-7/ADR xenograft after US+ADM treatment or ADM treatment on day 24; N = 6; data are represented as mean ± s.d; *P < 0.05; (**c**-**d**) Detecting ZEB1 expression in respective tumor tissue by western blot (**c**) and immunochemistry (**d**, scale bar = 10 μm); N = 6; data are represented as mean ± s.d; *P < 0.05; (**e**-**g**) Detecting P-gp expression in respective tumor tissue by western blot (**e**), immunochemistry (**f**, scale bar = 10 μm), and immunofluorescence (**g**, scale bar = 100 μm); N = 6; data are represented as mean ± s.d; *P < 0.05; (**h**) Illustration of reversal of MDR mediated by US exposure in MDR cancer cells; Ultrasound exposure increases miR-200c/34a expression by promoting ROS generation. MiR-200c/34a overexpression directly or indirectly inhibits ZEB1 and P-gp expression. Down-regulation of ZEB1 in turn decreases its transcriptional repression on miR200c/34a. P-gp inhibition sensitizes MDR cells to MDR-associated drugs and increases the cytotoxicity of these chemotherapeutics

To further confirm the relationship between US exposure, ROS, ZEB1, miR-200c/34a and P-gp, we analyzed their expression in isolated tumors from MCF-7/ADR xenograft nude mice. Consistent with the results in vitro, ROS activity in the US+ADM group was significantly higher than that in the ADM group (*P* < 0.05; Fig. 7a). Furthermore, qRT-PCR assays indicated miR-200c/34a expression level were significantly higher in the US+ADM group than ADM group (*P* < 0.05; Fig. 7b). Immunohistochemistry and western blotting showed that the expression of ZEB1 was significantly lower in US+ADM group, compared to ADM group (*P* < 0.05; Fig. 7c and d; respectively). Moreover, P-gp expression was significantly lower in US+ADM group than that in ADM group (*P* < 0.05; Fig. 7e, f and g; respectively).

### The effect of US exposure on drug-sensitive t cells

We also explored the effect of US exposure on drug-sensitive cancer cells using MCF-7 and HEPG2 cell lines. Similar to our observations on MCF-7/ADM and HUVEC cells, cell viability was not significantly affected in neither MCF-7 cells nor HEPG2 cells using ultrasound acoustic intensity ≤0.74 W/cm^2^ exposure (*P* < 0.05, ≥0.74 W/cm2, respectively; Additional file 7: Figure S5C). The IC50 dosage of ADM for MCF-7 and HEPG2 cells was 1.82 ± 0.34 and 1.68 ± 0.23 μg/ml, respectively. The IC50 dosage of ADM and 0.74 W/cm^2^ US was used in the relevant experiments. There was almost no difference in IC50 of ADM between ADM + US treatment groups and ADM treatment groups (*P* > 0.05, respectively; Additional file 7: Figure S5A, B and Additional file 4: Table S2). Confocal microscopy images revealed US exposure has no effect on the quantity of ADM nuclei localization in MCF-7 or HEPG2 cells (Additional file 7: Figure S5D). Furthermore, ADM concentration in MCF-7 or HEPG2 cells was not significantly changed in 24 h after US exposure (*P* > 0.05, respectively; Additional file 7: Figure S5E), which is similar to our observations in HUVEC. Moreover, we investigated whether US induces P-gp down-regulation on these non-drug resistant cells by promoting ROS generation. The western blot analysis revealed that the P-gp expression levels were remarkably lowered in MCF-7, HEPG2 and HUVEC cells compared with the MDR variants (Additional file 8: Figure S6B and C). Expectedly, US exposure significantly increased ROS activity in MCF-7, HEPG2 and HUVEC cells (*P* < 0.05, respectively; Additional file 8: Figure S6A). However, we did not detect a significant reduction on P-gp expression of these cells 24 h after US exposure (*P* > 0.05, respectively; Additional file 8: Figure S6B and C). Similarly, when we added ADM to culture dishes in 24 h after cells were exposed to US or control treatment, ADM intracellular concentration was not significantly increased in US groups, compared to control groups (*P* > 0.05, respectively; Additional file 8: Figure S6E). We found that sonoporation effect of US induced transient membrane perforation on MCF-7, HEPG2 and HUVEC cells, which almost disappeared in 60 min after US exposure (Additional file 8: Figure S6D). When we added ADM to culture dishes at 5 min after US exposure, ADM was transiently and mildly accelerated into MCF-7, HEPG2 and HUVEC cells (Additional file 8: Figure S6F). However, in 60 min post-treatment, ADM concentrations in US+ADM group were not significantly higher than that in ADM group (*P* > 0.05, respectively; Additional file 8: Figure S6F). Collectively, our results suggested US exposure could not significantly affect cytotoxicity of ADM or P-gp expression for non-drug resistant cells.

## Discussion

In this study, we found that 0.74 W/cm^2^ US exposure increased ADM intracellular concentration and enhanced ADM cytotoxicity against MDR cancer cells both in vitro and in vivo. Compared with ADM treatment alone, combining ADM and US exposure reduced tumor growth rate and improved long-term prognosis in MCF-7/ADR xenograft mice with no obvious increase in systemic toxicity. Mechanistically, US exposure promoted intracellular ROS generation and miR-34a/miR-200c expression. US-induced miR-34a/miR-200c and ZEB-1 formed a double-negative feedback loop to regulate P-gp expression and MDR phenotype.

In this study, we proposed US exposure as a novel treatment method for overcoming MDR without obvious side effects, and our results have confirmed this assumption. Our in *vitro* and in *vivo* data showed that 0.74 W/cm^2^ US exposure significantly increased intracellular ADM concentrations in ADM-resistant cells. In addition, we observed that a combination of ADM and US exposure resulted in a enhanced inhibition of apoptosis and proliferation in ADM-resistant cells compared with ADM treatment alone. More importantly, the combination of ADM and US exposure remarkably decreased tumor volume and improved prognosis in MCF-7/ADR xenograft mice. Our results are consistent with previous in vitro studies in which US exposure significantly increased the antitumor effect of ADM in neuroblastoma and ovarian MDR-variant cell lines [32, 33]. Particularly noteworthy, US exposure has several advantages over classical P-gp inhibitors. First, in contrast to chemical approach, US exposure reduced nonselective action on P-gp expressed in normal tissues by accurately targeting tumors, thus avoiding the systemic side-effects of classical P-gp inhibitors. This could be partly supported by the result in our experiments which showed that the combination of ADM and US exposure did not result in elevated deaths or obvious body weight loss amongst the tumor-bearing mice. This improvement is especially relevant for treating localized solid tumors. Moreover, because US treatment is a physical energy, the harmful interaction between P-gp inhibitors and other chemotherapy drugs can be avoided. All of these findings indicate that US exposure is a targeted, efficient, and safe treatment for cancer MDR.

The current study also demonstrated that increased ADM concentrations and reversal of MDR by US exposure was mainly due to decreased expression of P-gp expression. Previous studies have reported that US exposure temporarily increased intracellular drug retention in drug-sensitive cells [34]. In this study, we also observed that intracellular ADM concentrations of MDR cells increased mildly and temporarily when ADM administration was performed immediately after US exposure. Nonetheless, when ADM administration was performed 24 h after US exposure, substantially increased ADM concentrations could be stably maintained for more than 12 h. Further study showed that the short-term effects of US exposure mainly can be ascribed to elevated cell membrane permeability caused by the sonoporation effect, whereas long-term effects resulted from transcriptional repression of P-gp expression. Compared with the sonoporation effect, down-regulation of P-gp yielded greater ADM accumulation over a longer duration. Therefore, it is reasonable to deduce that down-regulation of P-gp expression may be the main mechanism by which US exposure increased ADM accumulation in MDR cancer cells. Overexpression of the membrane drug efflux pump P-gp is one of the major mechanisms by which cancer cells develop MDR. The findings that US irradiation reduced P-gp expression further suggest that US irradiation may be a potential anti-MDR treatment. Interestingly, as a promising strategy, transcriptional repression is not only effective, but also enables the prevention of P-gp expression during the progression of disease [35]. It has been noted that in some tumors, P-gp expression is low before exposure to chemotherapy drugs, but increases after chemotherapy and eventually results in MDR [36]. Future studies should determine whether US irradiation started during the early stage of chemotherapy could prevent the occurrence of the MDR phenotype and improve the efficacy of treatment.

In this study, we revealed that the ability of US irradiation to repress P-gp expression might be based on the generation of ROS. It is known that US irradiation can promote ROS production as a consequence of the cavitation phenomena, which may result in ectopic expression of genes [37]. Moreover, previous studies also revealed evidence supporting the role of oxidative stress in down-regulating P-gp expression [38–41]. In accordance with previous studies [42], our immunofluorescence results showed that US exposure increased intracellular ROS production. More important, administration with NAC, a well-known ROS inhibitor, significantly blocked the US-mediated ROS generation, and almost abrogated US-induced P-gp inhibition. These findings suggest that decreased P-gp expression following US treatment might be mediated by elevated ROS.

MiR-200c and miR-34a could be induced by oxidative stress in several cell types, and are designated as oxidative stress-responsive miRNAs [30, 43, 44]. In this study, we found that US radiation increased mir-200c and miR-34a expression through oxidative signal pathway, which was responsible for P-gp down-regulation. Tumor suppressor miR-34a-5p is often down-regulated in drug-resistant cells [45, 46]. Generated together with miR-34a-5p, miR-34a-3p has a similar expression level and functional role in different cells and tumor samples [47–49]. We found a remarkable increase in miR-34a-3p expression after US exposure, whereas a modest increase in miR-34a-5p. We further demonstrated miR-34a-3p could inhibit P-gp expression by directly binding to P-gp three prime untranslated region (3’-UTR). Repressing miR-34a-3p expression attenuated US-induced P-gp down-regulation, indicating the involvement of miR-34a-3p in US-mediated MDR reversal. Additionally, miR-200c has been demonstrated to reverse P-gp-mediated MDR by blocking JNK2/c-Jun pathway [29, 50]. Consistent with previous studies, we also found overexpression of miR-200c reduced P-gp expression and combated MDR phenotype, while miR-200c inhibition reversed US-mediated P-gp down-regulation. These results indicate the involvement of miR-200c/34a in US-mediated MDR reversal, and that miR-200c/34a could be potential targets in treating drug-resistance.

It is known that oxidation stress alters miRNA expression by affecting their transcriptional activity [51]. The underlying mechanisms include well-characterized redox-responsive alterations in activity of transcriptional factors (TFs) [52]. ZEB1 is a well-described transcription repressor that is associated with mi-200c/34a down-regulation [53, 54]. Importantly, it has been discovered that oxidation stress could increase miR-200c expression by forming miR-200c/ZEB1 double negative feedback loop [30]. In keeping with previous studies, we found that US exposure triggered miR-200c/ZEB1 circuitry through oxidative pathway. Moreover, we also found that miR-34a and ZEB1 could also form a double negative feedback loop. The presence of mir-200c/34a/ZEB1 circuitry may convey robustness to anti-MDR effect of US and prevent the disturbance emanating from the cellular environment.

Furthermore, we found that US exposure had no effect on the cytotoxicity of ADM for MCF-7, HEPG2 and HUVEC cells. We further uncovered that US could increase ROS activity, but could not significantly decrease P-gp expression in these cells. Our results are similar to previous studies showing targeting P-gp was less effective in treating MCF-7 cells than that in treating MCF-7/ADR cells [55, 56]. These probably result from the rather low baseline level of P-gp expression in drug-sensitive cells, which decreases the silence efficiency [56].

The current study has some limitations. First, only an appropriate US intensity, instead of a therapeutic window of US intensity, was investigated. To improve the clinical feasibility of this treatment, future study is required to explore a therapeutic window by testing additional intensity gradients. In addition, although 0.74 W/cm^2^ US exposure didn’t increase ADM concentration in apoptotic cells in peritumor muscle tissue, the effects of appropriate US intensity on other normal tissues should be investigated in future studies. What’s more, oxidative stress induced p53 up-regulation and pRb de-phosphorylation are both participated in miR-200c expression, reinforcing miR-200c/ZEB1 circuitry [30]. Whether such mechanisms also play a role in US-induced miR-200c/34a/ZEB1 feedback loop should be elucidated by future studies.

## Conclusions

In conclusion, US exposure enhances ADM intracellular uptake and accumulation of MDR cancer cells in vivo and in vitro. The increased cellular uptake improved cytotoxicity in MCF-7/ADR and HEPG2/ADM cells. The anti-MDR effect of US is associated with P-gp down-regulation. US exposure increases ROS generation and activates a double feedback loop formed by miR-200c/34a/ZEB1, leading to P-gp inhibition. These findings suggest that US exposure could serve as a promising treatment for MDR. In the future, well-designed clinical studies are required to further evaluate the feasibility and efficacy of US-mediated reversal of cancer MDR.

## Additional files

Additional file 1: **Figure S1.** Diagrammatic representation of US application in vitro. Sterilized US transducer was immersed in the culture medium and about 10 mm above the cell layer. (PDF 131 kb)

Additional file 2: **Table S1.** The primers used in this study. (DOCX 17 kb)

Additional file 3: **Figure S2.** (A-B) Modulation by US exposure of the sensitivity to ADM of MCF-7/ADR cells (A) and HEPG2/ADM cells (B); *N* = 3, data are represented as mean ± s.d; *P < 0.05 vs. ADM group; (C) Induction of apoptosis in two MDR cells was determined by flow cytometry after treatment with US+ADM or ADM. N = 3; data are represented as mean ± s.d; *P < 0.05. (PDF 265 kb)

Additional file 4: **Table S2.** The IC50 of ADM for MCF-7/ADR, HEPG2/ADM cells and their parental cells, and HUVEC. (DOC 30 kb)

Additional file 5: **Figure S3.** The dynamic change of ROS activity in drug-resistant cells after US stimulation. (A) Representative images of DCFH-DA staining in MCF-7/ADR cells exposed to US, (scale bar = 50 μm); (B) Quantitative analysis of ROS fluorescence intensity. *N* = 3; data are represented as mean ± s.d; ^*^P < 0.05 vs. 0 h. (PDF 321 kb)

Additional file 6: **Figure S4.** MiR-200c/34a modulated MDR phenotype. (A-B) MiR-200c/24a inhibition diminished the effect of US on enhancing ADM uptake (A) and ADM-inducing apoptosis (B) for MCF-7/ADR cells; *N* = 3; data are represented as mean ± s.d; ^*^P < 0.05; (C) MiR-200c/34a overexpression increased the intracellular ADM uptake in MCF-7/ADR cells; *N* = 3; data are represented as mean ± s.d; ^*^P < 0.05; (D) MiR-200c/34a overexpression increased the cytotoxicity of ADM in MCF-7/ADR cells; *N* = 3; data are represented as mean ± s.d; ^*^P < 0.05; (E) TUNEL staining detected the cell apoptosis of MCF-7/ADR cells transfected with miR-200c/34a mimics or control (scale bar = 50 μm); *N* = 3; data are represented as mean ± s.d; ^*^P < 0.05; (F) EdU staining detected the cell proliferation of MCF-7/ADR cells transfected with miR-200c/34a mimics or control (scale bar = 50 μm); *N* = 3; data are represented as mean ± s.d; ^*^P < 0.05. (PDF 332 kb)

Additional file 7: **Figure S5.** The effect of US exposure on the cytotoxicity of ADM for drug-sensitive cells. (A-B) The effects of US exposure on the sensitivity to ADM of MCF-7 cells (A) and HEPG2 cells (B); *N* = 3; data are represented as mean ± s.d; *P < 0.05 vs. ADM group; (C) Cell viability in MCF-7 and HEPG2 cells 24 h after US exposure with different acoustic intensities; *N* = 3; *P < 0.05 vs. 0 W/cm^2^ in MCF-7 cells; ^#^*P* < 0.05 vs. 0 W/cm^2^ in HEPG2 cells; (D) Images of intracellular ADM distribution in MCF-7, HEPG2 cells 24 h after US+ADM treatment or ADM treatment (scale bar = 10 μm); (E) Intracellular ADM concentration in MCF-7 and HEPG2 cells in 24 h after US+ADM treatment or ADM treatment; *N* = 3; *P < 0.05. (PDF 544 kb)

Additional file 8: **Figure S6.** US exposure has no effect on P-gp expression of MCF-7, HEPG2 and HUVEC cells. (A) Representative images and quantitative analysis of DCFH-DA staining in MCF-7, HEPG2 and HUVEC cells 24 h after treatment; *N* = 3; *P < 0.05 (scale bar = 50 μm); (B-C) Detecting P-gp expression of MCF-7 (B), HEPG2 and HUVEC cells (C) by western blotting in 24 h after US exposure; *N* = 3; data are represented as mean ± s.d; *P < 0.05; (D) Cell morphology and cytomembrane changes in MCF-7, HEPG2 and HUVEC cells after US exposure (scale bar = 5 μm); (E) The dynamic change of ADM concentration in MCF-7, HEPG2 and HUVEC cells treated with US+ADM or ADM alone. ADM was added to the mediums in 24 h after exposure to US; *N* = 3; data are represented as mean ± s.d; *P < 0.05; (F) The dynamic change of ADM concentration in MCF-7, HEPG2 and HUVEC cells treated with ADM immediately after US exposure; *N* = 3; data are represented as mean ± s.d; *P < 0.05. (PDF 874 kb)

## Abbreviations

3’ UTR: Three prime untranslated region
ABCB-1: ATP binding cassette subfamily B member 1
ADM: Adriamycin
CHIP: Chromatin immunoprecipitation
DCFH-DA: Dichlorofluorescein-diacetate
FBS: Fetal bovine serum
GDMLAC: Guangdong Medical Laboratory Animal Center
HUVEC: Human umbilical vein endothelial cell
IC50: Half-inhibitory concentration
L: length
MDR: Multidrug resistance
MiRs: MicroRNAs
NAC: N-acetyl-L-cysteine
P-gp: P-glycoprotein
Q-PCR: Quantitative polymerase chain reaction
ROS: Reactive oxygen species
TF: Transcriptional factor
TUNEL: Terminal deoxynucleotidyl transferase dUTP nick end labeling
US: Ultrasound
W: width
