# Supplementary figures and images for: The effects of ultrasound exposure on P-glycoprotein-mediated multidrug resistance in vitro and in vivo
Source: J Exp Clin Cancer Res. 2018 Sep 19;37:232. doi: 10.1186/s13046-018-0900-6 (PMC6149229; doi:10.1186/s13046-018-0900-6)

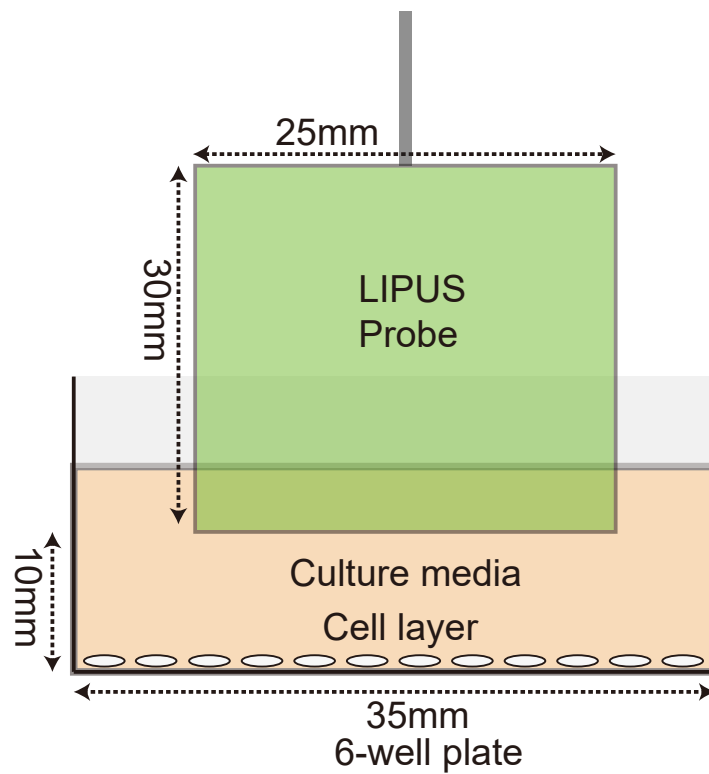

**Fig. S1**

Supplement: Supplementary file 1 — Figure S1. Diagrammatic representation of US application in vitro. Sterilized US transducer was immersed in the culture medium and about 10 mm above the cell layer. (PDF 131 kb) [file 13046_2018_900_MOESM1_ESM.pdf]

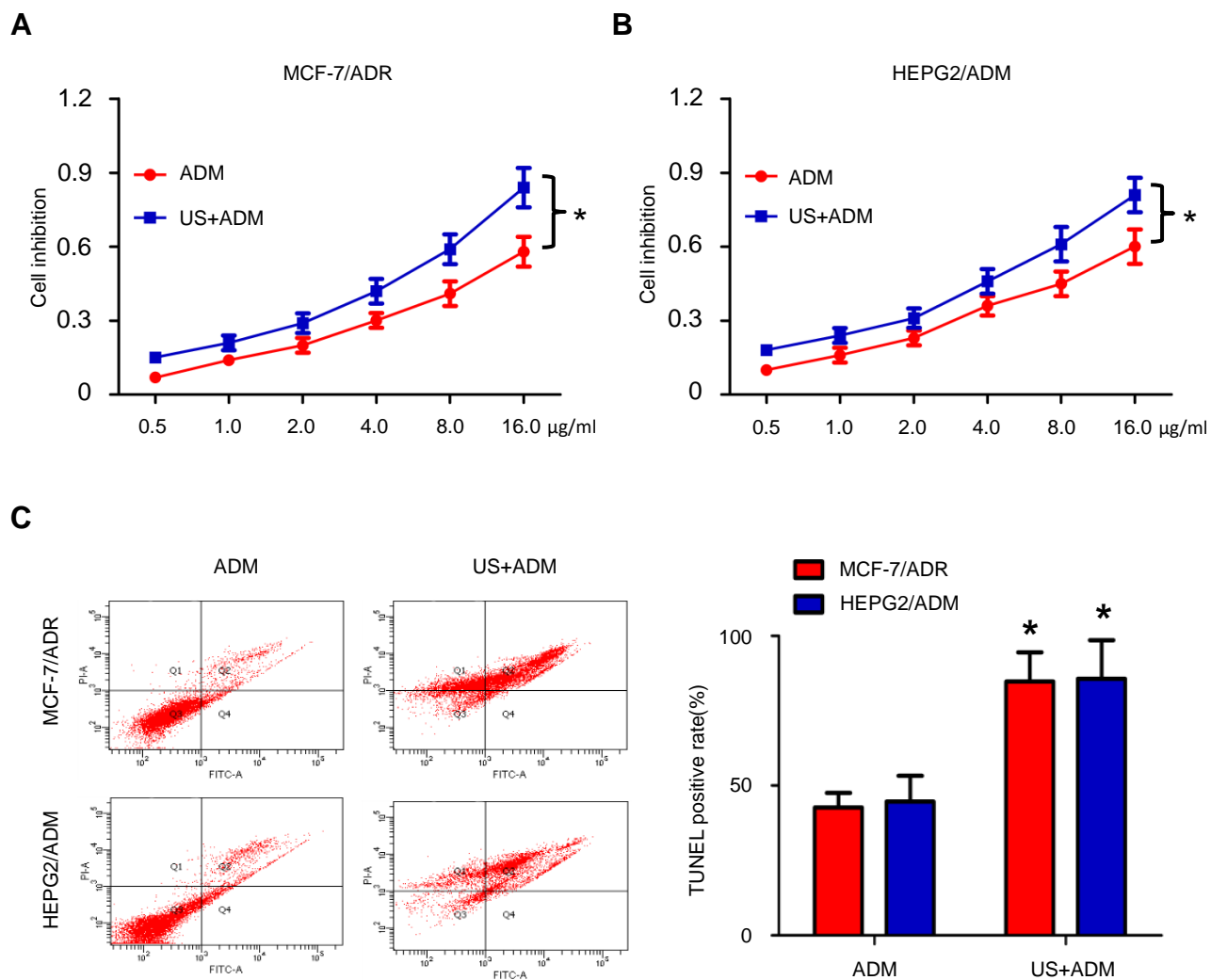

**Fig. S2**

Supplement: Supplementary file 3 — Figure S2. (A-B) Modulation by US exposure of the sensitivity to ADM of MCF-7/ADR cells (A) and HEPG2/ADM cells (B); N = 3, data are represented as mean ± s.d; *P < 0.05 vs. ADM group; (C) Induction of apoptosis in two MDR cells was determined by flow cytometry after treatment with US+ADM or ADM. N = 3; data are represented as mean ± s.d; *P < 0.05. (PDF 265 kb) [file 13046_2018_900_MOESM3_ESM.pdf]

**A**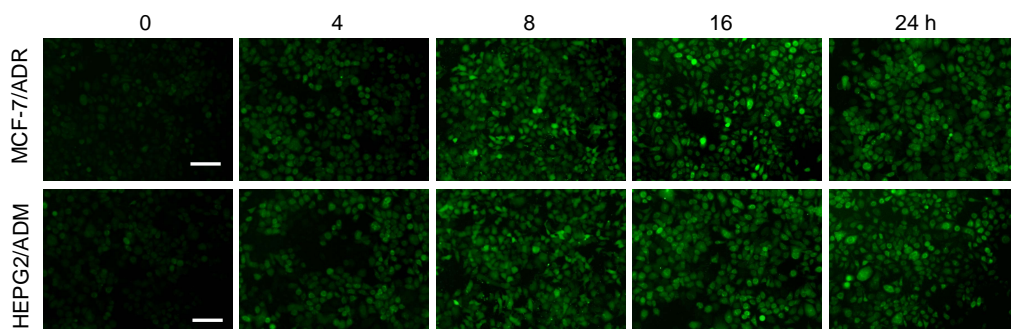**B**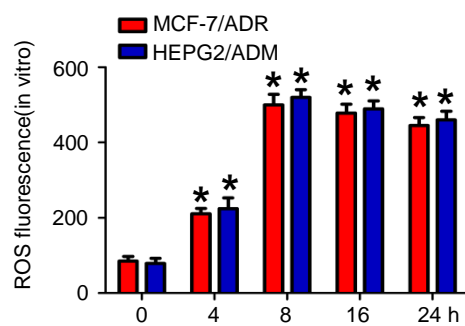**Fig. S3**

Supplement: Supplementary file 5 — Figure S3. The dynamic change of ROS activity in drug-resistant cells after US stimulation. (A) Representative images of DCFH-DA staining in MCF-7/ADR cells exposed to US, (scale bar = 50 μm); (B) Quantitative analysis of ROS fluorescence intensity. N = 3; data are represented as mean ± s.d; *P < 0.05 vs. 0 h. (PDF 321 kb) [file 13046_2018_900_MOESM5_ESM.pdf]

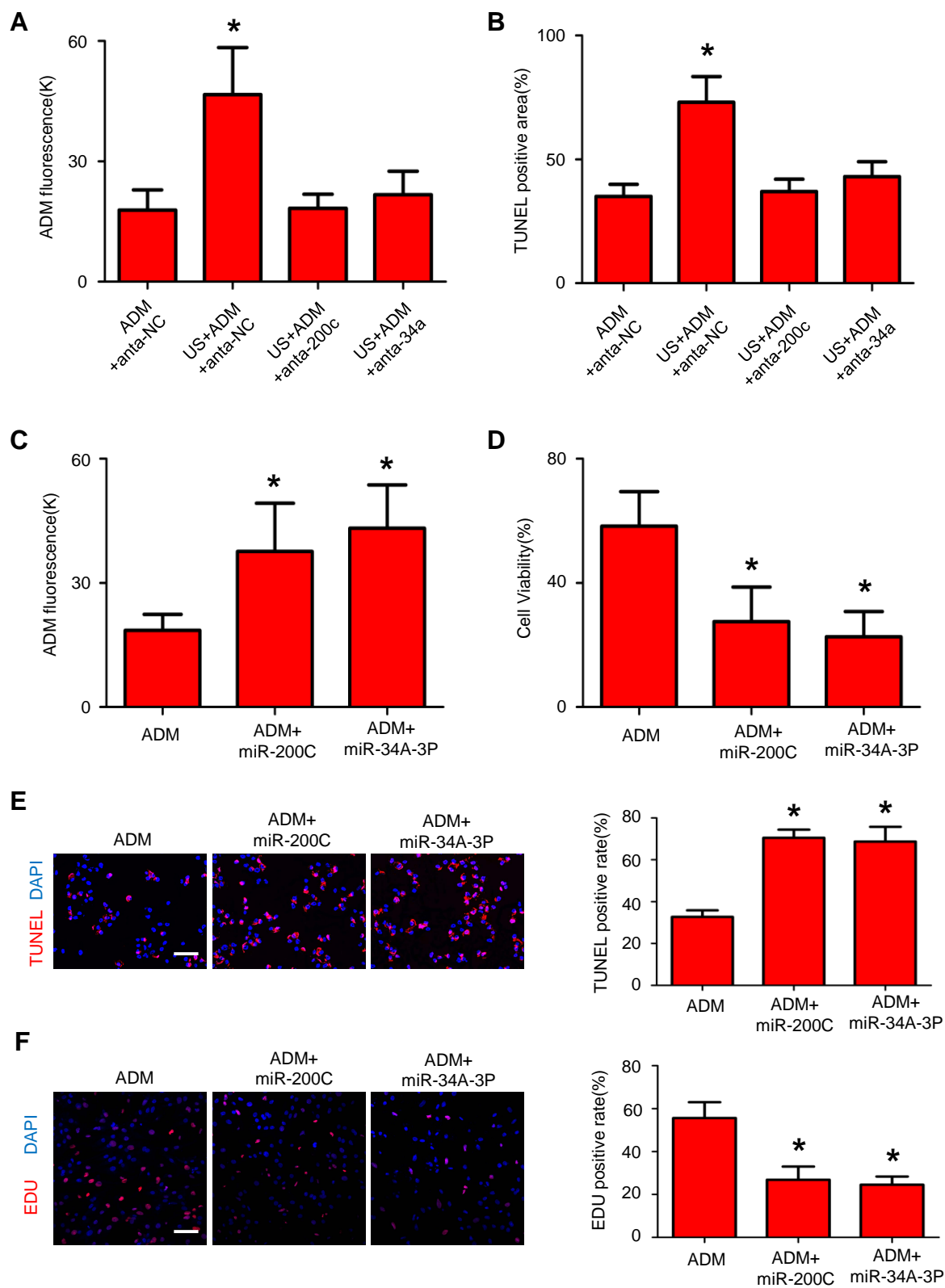

**Fig. S4**

Supplement: Supplementary file 6 — Figure S4. MiR-200c/34a modulated MDR phenotype. (A-B) MiR-200c/24a inhibition diminished the effect of US on enhancing ADM uptake (A) and ADM-inducing apoptosis (B) for MCF-7/ADR cells; N = 3; data are represented as mean ± s.d; *P < 0.05; (C) MiR-200c/34a overexpression increased the intracellular ADM uptake in MCF-7/ADR cells; N = 3; data are represented as mean ± s.d; *P < 0.05; (D) MiR-200c/34a overexpression increased the cytotoxicity of ADM in MCF-7/ADR cells; N = 3; data are represented as mean ± s.d; *P < 0.05; (E) TUNEL staining detected the cell apoptosis of MCF-7/ADR cells transfected with miR-200c/34a mimics or control (scale bar = 50 μm); N = 3; data are represented as mean ± s.d; *P < 0.05; (F) EdU staining detected the cell proliferation of MCF-7/ADR cells transfected with miR-200c/34a mimics or control (scale bar = 50 μm); N = 3; data are represented as mean ± s.d; *P < 0.05. (PDF 332 kb) [file 13046_2018_900_MOESM6_ESM.pdf]

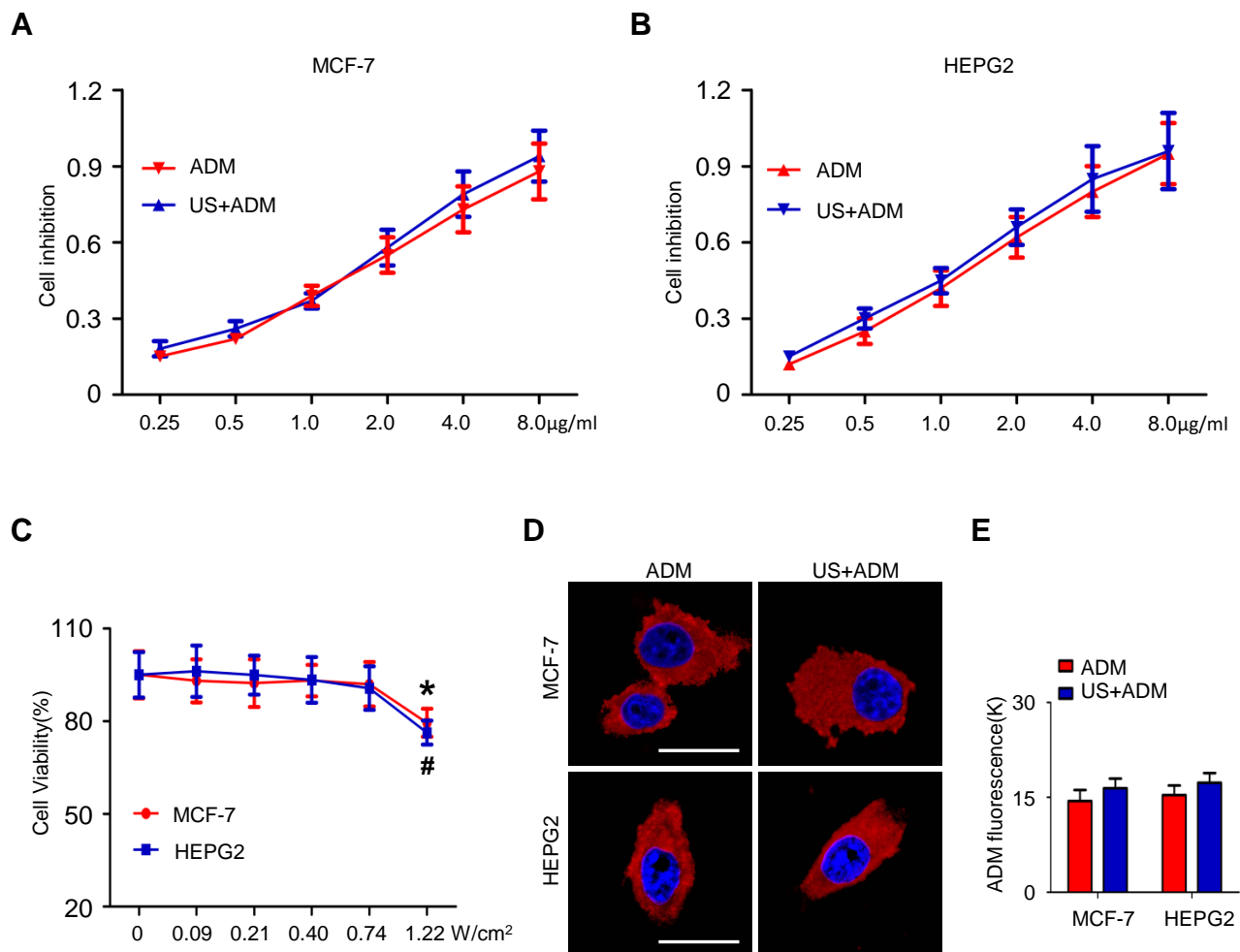

**Fig. S5**

Supplement: Supplementary file 7 — Figure S5. The effect of US exposure on the cytotoxicity of ADM for drug-sensitive cells. (A-B) The effects of US exposure on the sensitivity to ADM of MCF-7 cells (A) and HEPG2 cells (B); N = 3; data are represented as mean ± s.d; *P < 0.05 vs. ADM group; (C) Cell viability in MCF-7 and HEPG2 cells 24 h after US exposure with different acoustic intensities; N = 3; *P < 0.05 vs. 0 W/cm2 in MCF-7 cells; #P < 0.05 vs. 0 W/cm2 in HEPG2 cells; (D) Images of intracellular ADM distribution in MCF-7, HEPG2 cells 24 h after US+ADM treatment or ADM treatment (scale bar = 10 μm); (E) Intracellular ADM concentration in MCF-7 and HEPG2 cells in 24 h after US+ADM treatment or ADM treatment; N = 3; *P < 0.05. (PDF 544 kb) [file 13046_2018_900_MOESM7_ESM.pdf]

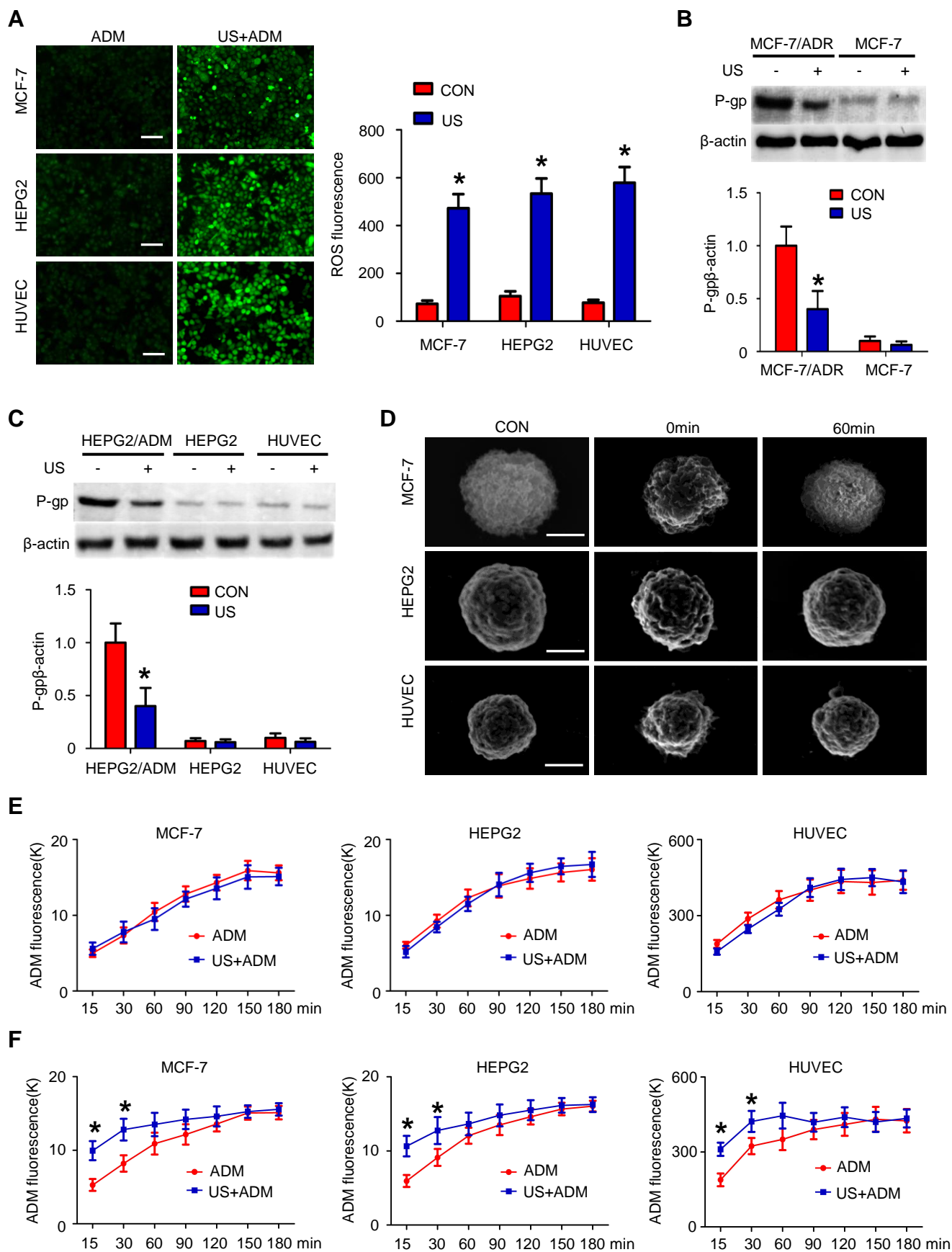

**Fig. S6**

Supplement: Supplementary file 8 — Figure S6. US exposure has no effect on P-gp expression of MCF-7, HEPG2 and HUVEC cells. (A) Representative images and quantitative analysis of DCFH-DA staining in MCF-7, HEPG2 and HUVEC cells 24 h after treatment; N = 3; *P < 0.05 (scale bar = 50 μm); (B-C) Detecting P-gp expression of MCF-7 (B), HEPG2 and HUVEC cells (C) by western blotting in 24 h after US exposure; N = 3; data are represented as mean ± s.d; *P < 0.05; (D) Cell morphology and cytomembrane changes in MCF-7, HEPG2 and HUVEC cells after US exposure (scale bar = 5 μm); (E) The dynamic change of ADM concentration in MCF-7, HEPG2 and HUVEC cells treated with US+ADM or ADM alone. ADM was added to the mediums in 24 h after exposure to US; N = 3; data are represented as mean ± s.d; *P < 0.05; (F) The dynamic change of ADM concentration in MCF-7, HEPG2 and HUVEC cells treated with ADM immediately after US exposure; N = 3; data are represented as mean ± s.d; *P < 0.05. (PDF 874 kb) [file 13046_2018_900_MOESM8_ESM.pdf]
