# Supplementary material for: The effects of ultrasound exposure on P-glycoprotein-mediated multidrug resistance in vitro and in vivo
Source: J Exp Clin Cancer Res. 2018 Sep 19;37:232. doi: 10.1186/s13046-018-0900-6 (PMC6149229; doi:10.1186/s13046-018-0900-6)
Supplement: Supplementary file 2 — Table S1. The primers used in this study. (DOCX 17 kb) [file 13046_2018_900_MOESM2_ESM.docx]

Additional file 2: **Table S1.** The primers used in this study.

| Primers name |  | Sequence (5’-3’) |
| --- | --- | --- |
| ABCB1 | + | 5’-TCT GGAGGA AGA CAT GAC CAG GTA-3’ |
|  | - | 5’-GGCACC AAA ATG AAA CCT GAA TGT-3’ |
| MRP | + | 5’-CGTGCTGAAGGCCTGTATCC-3’ |
|  | - | 5’-GCTTCTTGGCGGACTTTGG-3’ |
| LRP | + | 5’- TTTCTGACGGCAACTTCAAC-3’ |
|  | - | 5’- AGTCCAATGTCCAGCCCAT-3’ |
| BCRP | + | 5’-GCATCGATCTCTCACCCTGG-3’ |
|  | - | 5’- ATTGCTGCTGTGCAACAGTG-3’ |
| β-actin | + | 5’- CTG GAA CGG TGA AGG TGA CA-3’ |
|  | - | 5’-AAG GGA CTT CCT GTA ACA ATG CA-3’ |
| miR-200c | + | 5′-AGCGGTAATACTGCCGGGTA-3′ |
|  | - | 5′-GTGCAGGGTCCGAGGT-3′ |
| miR-34a-3p | + | 5′-CTCGCTTCGGCAGCACA -3′ |
|  | - | 5′-AACGCTTCACGAATTTGCGT-3′ |
| miR-92a | + | 5’-TCTACACAGGTTGGGATCGG-3’ |
|  | - | 5’-CGGGACAAGTGCAATACCATA-3’ |
| miR-200b | + | 5’-CGCAGCAGTGGAACCTGT-3’ |
|  | - | 5’-GTGAGGAGGTGCTGGGATG-3’ |
| miR-34a-5p | + | 5’-GGTGGCAGTGTCTTAGCT-3’ |
|  | - | 5’-CAGTGCGTGTCGTGGAGT-3’ |
| miR-210 | + | 5′-CTGTGCGTGTGACAG-3′ |
|  | - | 5′-GTGCAGGGTCCGAGGT-3′ |
| U6 snRNA | + | 5′-CTCGCTTCGGCAGCACA -3′ |
|  | - | 5′-AACGCTTCACGAATTTGCGT-3′ |
| luciferase ABCB1 | + | 5′- GTCATCTTGTCCAAACTGCCTGTG-3′ |
|  | - | 5′-ACATGAAAGTTTAGTTTTATTATAGACACTT-3′ |
| luciferase ZEB1 | + | 5′-AAAAATCCGGGTGTGCCTGA-3′ |
|  | - | 5′-AACTGCTTTCTACTGCTCTG-3′ |
| CHIP miR-200c | + | 5’-CTATGGCAGGAGGACACA-3’ |
|  | - | 5’-AGATTCCACGGCCTAGAG-3’ |
| CHIP miR-34a | + | 5’-CAGCAACAGGTGTAGGAA-3’ |
|  | - | 5’-CCTATTCTCACAGGTGTCT-3’ |
