# Supplementary material for: The effects of ultrasound exposure on P-glycoprotein-mediated multidrug resistance in vitro and in vivo
Source: J Exp Clin Cancer Res. 2018 Sep 19;37:232. doi: 10.1186/s13046-018-0900-6 (PMC6149229; doi:10.1186/s13046-018-0900-6)
Supplement: Supplementary file 4 — Table S2. The IC50 of ADM for MCF-7/ADR, HEPG2/ADM cells and their parental cells, and HUVEC. (DOC 30 kb) [file 13046_2018_900_MOESM4_ESM.doc]

Additional file 4: **Table S2.** The IC50 of ADM for MCF-7/ADR, HEPG2/ADM cells and their parental cells, and HUVEC.

| Groups | ADM | US+ADM |
| --- | --- | --- |
| MCF-7/ADR | 12.19±1.65 | 7.29±0.98***** |
| HEPG2/ADM  MCF-7  HEPG2  HUVEC | 10.26±1.29  1.82±0.34  1.68±0.23  45.28±4.97 | 6.42±1.31*****  1.69±0.29  1.57±0.35  43.75±4.46 |

(N=3, **P* < 0.05, Mean ± SD, µg/ml)
